# Micronutrients in Pregnancy after Bariatric Surgery: A Narrative Review

**DOI:** 10.3390/jcm12165429

**Published:** 2023-08-21

**Authors:** Irene Bretón, María D. Ballesteros-Pomar, Alfonso Calle-Pascual, Luis Antonio Alvarez-Sala, Miguel Angel Rubio-Herrera

**Affiliations:** 1 Department of Endocrinology and Nutrition, Hospital General Universitario Gregorio Marañón, Instituto de Investigación Sanitaria Gregorio Marañón, 28007 Madrid, Spain; 2 Department of Medicine, Facultad de Medicina, Universidad Complutense, 28040 Madrid, Spain; 3 Department of Endocrinology and Nutrition, Complejo Asistencial Universitario de León, 24008 León, Spain; 4 Department of Endocrinology and Nutrition, Hospital Clínico Universitario San Carlos, Instituto de Investigación Sanitaria del Hospital Clínico San Carlos, 28040 Madrid, Spain; 5 Centro de Investigación Biomédica en Red de Diabetes y Enfermedades Metabólicas Asociadas (CIBERDEM), 28029 Madrid, Spain; 6 Department of Internal Medicine, Hospital General Universitario Gregorio Marañón, 28007 Madrid, Spain

**Keywords:** bariatric, pregnancy, micronutrient deficiency

## Abstract

Bariatric surgery is increasingly used in women of childbearing age due to the rising prevalence of obesity and the effectiveness and availability of this treatment. Pregnancy in women with previous bariatric surgery deserves special attention. Weight loss induced by surgery reduces the risks that obesity poses to pregnancy. But on the other hand, decreased intake and malabsorption may increase the risk of malnutrition and micronutrient deficiency and negatively affect maternal and foetal health. The aim of this narrative review is to provide an updated analysis of the impact of different bariatric surgery techniques on mineral and micronutrient nutritional status during pregnancy and the possible effect on maternal–foetal health.

## 1. Introduction

Obesity is the most common metabolic disease in our environment and is associated with numerous medical and psychosocial complications and a clear deterioration in the quality of life. People with obesity have an increased risk of overall mortality and of developing other pathologies, such as type 2 diabetes, arterial hypertension, dyslipidaemia, respiratory diseases, and neoplasms, among others [1,2].

Obesity directly affects reproductive function in both males and females through complex and not fully understood mechanisms. In women, obesity increases the risk of infertility and is a poor prognostic factor when assisted reproduction techniques are used [3,4]. Both pregestational maternal obesity and excessive weight gain are associated with an increased risk of maternal–foetal complications, both short- and long-term [5,6,7] (Table 1). The risk of developing these complications is high, more than double that of normal-weight women [8]. It is estimated that 24.9% of the risk of any complication can be attributed to maternal overweight, and this attributable risk reaches 31.6% in the case of a large-for-gestational-age newborn (LBW) [8]. In morbidly or extremely obese women, the risk of complications during pregnancy and delivery is even higher [9,10].

Bariatric surgery results in significant and sustained weight loss in people with severe obesity and can decrease the risk of mortality and induce the remission or improvement of most comorbidities. The increase in the prevalence of obesity and the efficacy of bariatric surgery are leading to the increasing use of this treatment, especially in women of childbearing age.

Pregnancy in women with previous BS deserves special consideration. Weight loss induced by surgery reduces the risks that obesity implies for pregnancy. However, due to its effect on nutrient intake and absorption, it can also have adverse consequences on maternal and foetal health. Among them, vitamin and mineral deficiencies are especially frequent and require a protocolised evaluation and treatment.

The aim of this narrative review is to provide an updated analysis of the impact of different bariatric surgery techniques on mineral and micronutrient nutritional status during pregnancy and the possible effect on maternal–foetal health.

## 2. Bariatric Surgery and Pregnancy

**Figure 1:**
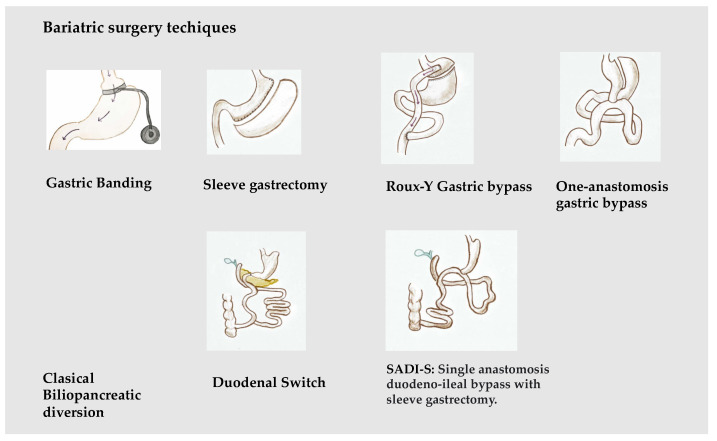
Main bariatric surgical techniques.

Bariatric surgery (BS) includes a set of surgical techniques used in patients with severe forms of obesity, with an aim to achieve weight loss, maintained over time, in order to improve associated diseases and the quality of life. Various bariatric surgery techniques have been described [11]. Based on their main mechanism of action, they are usually classified into three main groups: restrictive: vertical banded gastroplasty, adjustable gastric banding, and gastrectomy; mixed: gastric bypass; and malabsorptive: biliopancreatic diversion and its variants. The most frequently used BS procedures are Roux-Y gastric bypass (RYGB) and sleeve gastrectomy (SG); biliopancreatic diversion (BPD) and gastric banding (GB), widely used a decade ago, have been displaced by the first two [12] (Figure 1).

**Figure 2:**
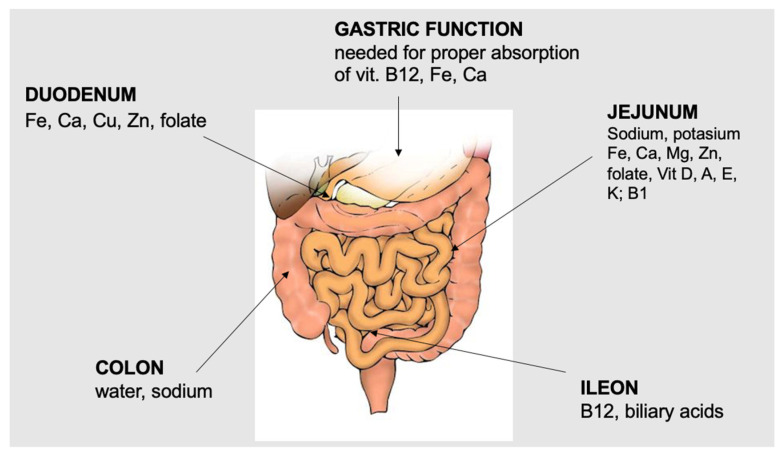
Main sites of absorption of minerals and micronutrients.

The mechanisms by which BS induces weight loss and improvements in metabolic diseases are complex and not fully understood [13,14]. Decreased intake and malabsorption, when present, are ultimately responsible for weight loss and improvements in obesity-related complications, but surgery is also able to induce changes in digestive hormones, such as ghrelin or GLP-1, among others, which are involved in the regulation of energy expenditure, insulin sensitivity and secretion, modification of the microbiota [15], or the physiology of bile acids [16,17]. Several studies have shown that, in patients with severe obesity, bariatric surgery decreases the risk of mortality and can achieve the remission or improvement of most comorbidities [18,19]. However, these procedures are not without risks. Among these, vitamin and mineral deficiencies are particularly common and require protocolised assessment, preventive supplementation, and treatment [20,21]. Figure 2 shows the preferred sites of the absorption of minerals and micronutrients.

The risk of mineral and micronutrient deficiencies after bariatric surgery depends on the patient’s dietary intake and on the anatomical and functional changes induced by the surgery itself. Malabsorption is frequent in techniques that exclude the duodenum and first jejunal loops and/or with a long biliopancreatic limb. It should be noted that in some techniques that are usually not considered “malabsorptive”, such as Roux-Y gastric bypass, the length of the limbs can vary considerably, and the biliopancreatic limb can be 150–200 cm or more. In these cases, although the malabsorption of proteins and other macronutrients is not frequent, the risk of a deficiency of micronutrients absorbed in the duodenum and first jejunal loops, such as iron, calcium, or copper, is greatly increased. Steatorrhea further decreases the absorption of calcium and liposoluble vitamins. Bariatric surgery induces changes in several gastrointestinal hormones, such as GLP-1, which has been linked to decreased food intake, improved glucose metabolism, and other lesser-known effects, such as the modulation of the sense of taste [22]. No specific role in micronutrient absorption after BS has been described so far. Table 2 summarises the main mechanisms leading to micronutrient deficiencies following different bariatric surgery techniques.

Scientific societies have provided recommendations for clinical and nutritional follow-up after bariatric surgery and proposals for preventive micronutrient supplementation, according to the type of surgical technique [23,24] (Table 3).

The increasing number of BS procedures being performed today, especially in women of childbearing age, makes post-surgery gestation a topic of great interest. Several systematic reviews and meta-analyses that evaluate the effect of BS on maternal and foetal outcomes have been published. Bariatric surgery can decrease the risk of obesity-related complications in pregnancy [25,26,27]. However, it may also have adverse consequences on maternal–foetal health [28,29,30,31,32,33,34]. Galazis et al. published a meta-analysis that includes 17 studies and provides results depending on the characteristics of the control group [25]. Thus, it is observed that the benefits of BS in reducing the risk of complications are more evident when maternal–foetal outcomes are compared with those of women with severe obesity. When the control group includes women with obesity, but the BMI is adjusted to pre-pregnancy, a decreased risk of LGA, but an increased risk of preterm delivery and a small-for-gestational-age (SGA) newborn, is observed. In this case, no increase or a decrease in the risk of GD, preeclampsia, the need for caesarean section, or neonatal complications is observed. The meta-analysis performed by Kwong et al. provides data on the effect of BS on maternal–foetal complications when compared with a group with similar BMI before BS and before gestation. The results of this meta-analysis indicate that, when compared with women with equal pregestational BMI, prior BS decreases the risk of high birth weight and increases the risk of low birth weight (<2500 g), SGA, the need for caesarean section, and prematurity. No changes in the risk of GD or preeclampsia or neonatal complications were observed [27]. There was a significant increase in the risk of perinatal mortality, congenital anomalies, preterm birth, and neonatal ICU admission, with a birth weight more than 200 g lower than those born to mothers without prior BS [35].

There is little information on the most appropriate interval between bariatric surgery and pregnancy and on the risk of complications of early gestation [36,37,38]. Clinical guidelines recommend delaying pregnancy for 12–18 months after BS, until weight loss has stabilised and dietary intake is adequate, to prevent nutritional deficiencies [31,32,39,40]. Although studies that have evaluated early pregnancies have not consistently observed an increase in complications [36,37,41], a closer clinical follow-up is recommended in these cases.

## 3. Micronutrients, Pregnancy, and Bariatric Surgery

Maternal nutritional factors are of great importance for foetal development. Energy and nutrient requirements increase during gestation to allow for adequate embryonic and foetal development and the necessary changes in the mother for pregnancy and lactation. A balanced diet, which provides enough energy, usually contains enough essential micronutrients. However, during pregnancy, requirements are often not met by food-based diets, and in these situations, specific supplementation is necessary to prevent deficiencies [42]. Micronutrients are essential for foetal development (Table 4). There is no unanimous agreement on preventive supplementation during pregnancy, which will depend on the characteristics of the pregnant woman and the risk of deficiency in her environment [43,44,45,46,47].

Previous bariatric surgery, especially if there is significant malabsorption, can increase the risk of malnutrition and micronutrient deficiencies during pregnancy, with adverse consequences for the mother and newborn [29,30,31,32,33,34]. There are several factors that may favour the risk of micronutrient deficiency in pregnancy after bariatric surgery, including decreased intake, gastrectomy, and malabsorption, as has been described before. There are few data on dietary intake in pregnant women with previous barbaric surgery. Available information suggests that it may be insufficient in essential nutrients [44]. In general, malabsorptive techniques are associated with a higher risk of complications in pregnancy after BS, especially maternal anaemia and low-birth-weight babies [45]. Anyway, it should be noted that patients who undergo malabsorptive BS have a higher pre-surgical BMI and that pregnancy in women with a BMI over 50 kg/m^2^ presents a very high risk of complications [46]. There are other risk factors for micronutrient deficiency that should not be overlooked. Veganism increases the risk of vitamin B12, iron, and zinc deficiencies. Drug–nutrient interaction therapy should be considered; for example, proton-pump inhibitors can decrease vitamin B12, iron, and magnesium absorption. Smoking and consumption of alcoholic beverages, well-recognised causes of pregnancy complications and alterations in foetal development, can also induce micronutrient deficiencies [47]. The evaluation of the nutritional status of vitamins and minerals should consider the reference serum values during pregnancy [48]. 

This section describes the main micronutrient deficiencies in pregnancy after bariatric surgery. For each micronutrient, its physiology, its relationship with pregnancy, the effect of bariatric surgery, and data on its deficiency in pregnancy after bariatric surgery are described. 

### 3.1. Folate

Folic acid and folate are precursors of the coenzyme tetrahydrofolate, which is involved in the transfer of one-carbon groups in the metabolism of amino acids and nucleic acids. It also participates as a donor in the methylation of homocysteine to methionine. Folate deficiency alters DNA synthesis and cell division and induces megaloblastic anaemia, leukopenia, thrombopenia, and other alterations [49]. Folate stores are not high: the body’s folate content ranges from 5 to 10 mg [49]. The main dietary sources of folate are vegetables, fruits, cereals, eggs, and fortified foods. Absorption occurs preferentially in the proximal third of the small intestine, being lower in patients with atrophic gastritis or intestinal resection. Folate requirements range from 300 to 400 μg/day, according to various guidelines and recommendations [50]. It is recommended that women of childbearing age receive a dose of 400 mg, in addition to that provided by food [50].

Folic acid is a relevant micronutrient during pregnancy and is essential for neural tube development [51]. Folate requirements increase in pregnancy by 50% because of increased maternal plasma volume, uterine and placental size, and foetal development. Maternal folate intake is related to newborn weight [52]. Most organisations recommend a total folate intake of 600 μg/day [50]. Folate deficiency during pregnancy is associated with neural tube defects (NTDs), with clinical manifestations of varying degrees of severity. In the brain, it can cause anencephaly and encephalocele, situations incompatible with life. In the spinal cord, it causes spina bifida syndrome, an isolated cleft of the spine, meningocele, and myelo-meningocele. In 90% of cases, they appear as isolated malformations. Folate deficiency has also been linked to recurrent miscarriage, placental abruption, and prematurity [53], probably related to a toxic effect of homocysteine on the embryo and alterations in placental vascularisation [54]. Pregnant women with obesity are at increased risk of NTDs. Obesity may lead to decreased plasma folate levels and increased erythrocyte uptake [55]. Folate requirements during pregnancy are generally not met by the diet. Folate supplementation, alone or in combination with vitamins and minerals, reduces the risk of NTDs [56]. However, it has no clear effect on other malformations or on other clinical variables of pregnancy [57]. Most guidelines recommend preventive supplementation with 400 mcg/day, starting 4 weeks before conception and lasting until at least week 12 of gestation. In women at high risk of neural tube defects, including women with obesity, a higher dose of 1–4 mg/day is recommended [58]. It should be kept in mind that doses higher than 1 mg/day may mask symptoms of vitamin B12 deficiency. 

The prevalence of folate deficiency after bariatric surgery is highly variable in different series. Although folate absorption occurs primarily in the proximal intestine, there is an intestinal adaptation that allows it to be absorbed throughout the intestine after a bowel resection. For this reason, the prevalence of folate deficiency after BS is lower than for other water-soluble vitamins and is generally easily prevented with a multivitamin [59]. Anyway, deficiency has been reported in up to 44–65% of patients after restrictive techniques [60,61] and 8–47% after mixed techniques [59,62]. However, other authors have not observed deficiencies after RYGB or BPD [63]. A systematic review of the literature did not reveal an increase in folate deficiency after BS [64].

Data on the prevalence of folate deficiency throughout gestation after BS are very scarce. Very few cases of NTDs have been reported [65,66]. This is striking, considering the increasing number of such pregnancies and the fact that obesity itself increases the risk of NTDs. Regarding the prevalence of folate deficiency, the reference serum levels for pregnancy should be noted. In a prospective study of 49 patients with malabsorptive BS, 4% had levels below 2.52 ng/mL in the first trimester [67]. In another retrospective study analysing 39 pregnancies (including 9 miscarriages), decreased levels were observed in 16% of pregnancies in the first trimester [68]. In a retrospective multicentre study in Spain, folate deficiency was observed in 5.4% of pregnancies [69].

### 3.2. Vitamin B12

Vitamin B12 is present in animal tissues. Its absorption is very complex: it first needs to be freed from dietary proteins via the action of gastric acid and pepsin and then binds to haptocorrins, glycoproteins present in salivary and gastric secretions. The absorption of vitamin B12 occurs specifically in the distal ileum. Vitamin B12 is excreted via the bile and undergoes enterohepatic circulation [70]. Vitamin B12 deficiency results in megaloblastic anaemia, and, in severe cases, leukopenia and thrombopenia may occur. In addition, vitamin B12 deficiency has neurological effects, with paraesthesias and the involvement of the posterior cords of the spinal cord, which can sometimes occur in the absence of anaemia [70]. 

Vitamin B12 deficiency during pregnancy is associated with an increased risk of prematurity, miscarriage, intrauterine growth retardation and low birth weight, neural tube defects, and impaired cognitive development [71,72].

The complexity of vitamin B12 absorption makes vitamin B12 deficiency very common in any clinical situation involving the gastrointestinal tract, including bariatric surgery [73]. In mixed and malabsorptive techniques, deficiency is very common; it affects more than 75% of cases and increases as time passes after BS, when the body’s stores of this vitamin are depleted, so prophylactic supplementation is recommended [73,74]. In general, it is recommended that vitamin B12 be administered intramuscularly (1000 μg/30–90 days) [75,76]. Oral or sublingual administration at high doses (more than 350 mg/day) also normalises plasma levels [75]. 

Clinical vitamin B12 deficiency during pregnancy after BS is rare but can cause serious problems in the newborn or infant, even in the absence of maternal symptoms [77,78,79]. Vitamin B12 requirements during pregnancy or lactation after BS are not established. Guidelines recommend a similar pattern to that in the non-pregnant population [76]. A recent study evaluated the levels of several micronutrients in the cord blood of 56 NBs of mothers with a history of BS compared to a group of NBs of healthy mothers. In the case of vitamin B12, decreased levels were observed in 14% vs. 2% (*p* < 0.05) [80]. Another study evaluated plasma vitamin B12 levels in 150 pregnancies after different BS techniques. Vitamin B12 levels did not decrease throughout gestation. An asymptomatic decrease in plasma levels (<130 pg/mL) was observed in 11.7% in the BPD group, 15.6 after RYGB, and 11% after SG; in eight patients, it was below 100 pg/mL [81]. Preventive vitamin B12 supplementation recommendations should be followed after BS and maintained throughout pregnancy. The dose included in ordinary or usual pregnancy multivitamins is not sufficient. 

### 3.3. Thiamine

Thiamine is a water-soluble vitamin involved in carbohydrate metabolism, such as glycolysis, as a cofactor of the enzyme pyruvate dehydrogenase, and the pentose pathway. It is present in many foods and is absorbed primarily in the proximal small intestine. Body stores are very low, which increases the risk of clinical deficiency if intake is inadequate [82]. Thiamine deficiency is promoted by decreased intake, vomiting, intravenous glucose administration, refeeding, or ethanol intake and results in a clinical picture with ophthalmoplegia, nystagmus, confusion, and peripheral neuropathy. Laboratory data (plasma thiamine levels or transketolase activity) confirm the deficiency. Magnetic resonance imaging is of particular interest, as it characteristically identifies a hyperintense T2 signal in the periaqueductal white matter [83]. Treatment should be initiated immediately, even if it is based solely on clinical suspicion, by administering high doses of thiamine (300–500 mg/day parenterally), followed by 50–100 mg/day orally for months [84].

Thiamine requirements increase during pregnancy and lactation [85]. Hyperemesis gravidarum is a recognised cause of thiamine deficiency and is probably underdiagnosed [86]. The recommended daily intake during normal pregnancy and lactation is 1.4 mg/day [87].

Thiamine deficiency is a complication of bariatric surgery, which can have serious consequences and lead to irreversible neurological damage [88,89]. It has been described with all surgical techniques: most have been identified in patients with nausea and vomiting, poor oral tolerance, or poor compliance with supplementation. Diagnosis should be made early, as the delay can lead to irreversible neurological damage and, in the case of pregnancy, can have serious consequences for the mother and foetus [90]. It is recommended that all patients receive ≥12 mg/day of thiamine after BS [23]; supplementation should be maintained throughout life [91]. This dose should be increased in case of any risk of deficiency, such as anorexia, low intake, vomiting, or refeeding (50–300 mg/day). 

There are few data in the literature on thiamine deficiency during pregnancy in women with previous BS. So far, one case of clinical deficiency has been described in a post-RYGB pregnancy with hyperemesis gravidarum [92]. Asymptomatic low thiamine serum levels in the third trimester have been observed in 17% of patients, being more frequent in malabsorptive techniques [67]. In another study evaluating serum levels in 57 pregnancies, decreased serum levels were observed in 45.5, 15.4, and 20% in the three trimesters of gestation, respectively [93]. In both studies, normal ranges for the non-pregnant population were used.

### 3.4. Calcium and Vitamin D

Calcium is an essential nutrient in human physiology that is involved in several metabolic pathways. Vitamin D-dependent calcium absorption takes place mainly in the duodenum and first jejunal loops; calcium requirements depend on the nutritional status of this vitamin. Vitamin D has multiple functions in the body: it promotes the absorption of calcium, phosphorus, and magnesium in the intestine, which allows for adequate bone mineralisation, stimulates innate and adaptive immunity, inhibits cell proliferation, and stimulates cell differentiation. The Endocrine Society proposes a serum level >30 ng/mL [94]. 

Vitamin D deficiency (VDD) is common in the general population and in pregnancy and can have adverse consequences on maternal and foetal health [95]. Vitamin D is involved in placental function, and its deficiency has been linked to insulin resistance and GD and to preeclampsia [96]. It is also involved in the development of the foetal nervous system, immune function, and lung maturation. VDD has been associated with an increased risk of low birth weight in some studies, but others have not observed this association. A meta-analysis including 12 studies with 19,027 patients found that vitamin D below 20 ng/mL is associated with an increased risk of SGA (birth weight percentile < 10), with an OR of 1.41, 95% CI 1.14, 1.75, 1.75 [97]. Women with deficient VitD levels had a higher preeclampsia rate compared to women with replete VitD levels (OR 1.50, 95% CI 1.05–2.14) [98]. Vitamin D below 16 ng/dL has been associated with a higher risk of C-section, and a level below 14 ng/dL has been associated with a higher risk of prematurity [99].

Bariatric surgery can induce calcium deficiency due to low dietary intake and malabsorption. Vitamin D deficiency is very common in morbidly obese patients. Bariatric surgery, especially when malabsorptive techniques are used, favours vitamin D deficiency, which is observed in more than 70% of cases. Long-term follow-up studies have also shown that VDD prevalence is high and is generally associated with increased PTH levels, which may have consequences for bone health. A systematic review of the literature confirms that hyperparathyroidism persists in the long term, despite calcium and vitamin D supplementation [100]. Several prevention and treatment guidelines have been described, which in general include regular monitoring, calcium supplementation, especially in techniques that exclude the duodenum, and vitamin D supplementation at the necessary dose, depending on the surgical technique [101]. The ASMBS recommends supplementing all patients with calcium (BPD 1800–2400 mg/day; RYGB, SG, and GB 1200–1500 mg/day) and vitamin D to maintain plasma levels above 30 ng/mL, generally 3000 IU/day [23,75].

Vitamin D deficiency is prevalent in pregnancy after bariatric surgery, although data in the literature are limited. In a study conducted in Brazil in 46 pregnancies after RYGB, 70% had levels below 20 ng/mL in all three trimesters. Hypocalcaemia was reported in 15% of cases in the first and second trimesters and in 20% in the third trimester and an increased PTH in 32.6% of pregnancies in the third trimester [102]. These data were confirmed in another retrospective study evaluating 42 pregnant women with a history of RYGB, also conducted in Brazil, which observed an inadequacy of vitamin D levels of up to 90 [103]. Bariatric multivitamins can prevent vitamin D deficiency during pregnancy in women with previous RYGB [104] As bariatric surgery increases the risk of vitamin D deficiency, pregnancy in women with BS may pose a risk to bone health if not adequately supplemented. 

### 3.5. Vitamin A

Vitamin A, present in foods as retinol or retinyl esters or as provitamins in the form of carotenoids, plays an important role in cell growth and differentiation, vision, immunity, and reproduction. The main dietary sources are liver, dairy, and fish oils, as well as coloured vegetables in the case of provitamin A carotenoids. Vitamin A deficiency (VAD) is very prevalent in developing countries and is a public health problem [105]. A diagnosis is considered when plasma levels are below 20 μg/dL [106]. Retinol is bound to prealbumin and retinol-binding protein (RBP), and serum levels may not always indicate vitamin A nutritional status [107].

Vitamin A is an essential nutrient in embryonic and foetal development for lung and sense organ maturation [108]. VAD during pregnancy is associated with an increased risk of low birth weight, prematurity, lung disease (bronchopulmonary dysplasia), an increased risk of infection in the newborn, and mortality in the neonatal period. In addition, vitamin A deficiency decreases iron mobilisation and increases the risk of anaemia [109]. It is estimated that there are approximately 19 million pregnant women with vitamin A deficiency [110]. The WHO recommends universal vitamin A supplementation during pregnancy in regions with a high prevalence of deficiency and night blindness [111]. During gestation, an intake of 700 μg/day is recommended [112]. High intakes of vitamin A (>3000 μg/day or >10,000 IU/day) during pregnancy are associated with an increased risk of malformations and should be avoided [112]. 

Bariatric surgery is nowadays one of the leading causes of clinical vitamin A deficiency in developed countries. The prevalence of decreased vitamin A serum levels in malabsorptive techniques can reach 60% [113]. In gastric bypass, a prevalence of 11% has been reported, and it is associated with visual symptoms such as xerophthalmia or night vision impairment [114]. In a prospective study in GBP patients, decreased levels were observed in 37.5%, 50.8%, and 52.9% preoperatively, at 30 days, and at 180 days, respectively [115]. Clinical vitamin A deficiency, manifested by visual and skin changes, is less common. Several clinical cases have been reported, especially after BPD [116,117,118,119,120]; the prevalence of clinical deficiency can reach 2.8–10% in some series. The ASMBS recommends supplementation with 5000–10,000 IU/day in malabsorptive BS [6,23,75].

Vitamin A deficiency may have adverse effects on pregnancy after bariatric surgery [121]. Some cases of malformations, such as microphthalmia, and other foetal complications secondary to maternal VAD have been reported. The first case of clinical vitamin A deficiency during gestation, 13 years after BPD, was published in 2002 [122]. Subsequently, other cases were published [123,124,125], most of them in patients with malabsorptive bariatric surgery and poor clinical follow-up. Regarding the adequacy of serum levels to reference values, Studies have shown discordant results regarding the adequacy of vitamin A serum levels. In a study conducted in Brazil in 30 pregnant women after RYGB, the prevalence of VAD (<30 μg/dL) reached 90%; 86.7% developed night blindness. No association was observed with maternal anaemia, which affected 73.3% of women. No data on pre-pregnancy VAD are provided in this study [126]. The prevalence of decreased serum levels in other countries has ranged from 20 to 60. No relationship has been observed between the different techniques or with maternal and foetal complications [93]. In a case–control study in France, a higher percentage of vitamin A levels in cord blood below the 2.5th percentile was observed in pregnancies after RYGB. It should be noted that in this study, the percentage of low birth weight for gestational age was high (23% vs. 3% in the control group) [80].

### 3.6. Vitamin E

Vitamin E is a fat-soluble vitamin with an antioxidant function and is mainly found in animal fats and oils. Its deficiency, which is very rare, leads to ataxia and other neurological symptoms, as well as to increased red blood cell fragility and haemolytic anaemia [127,128].

Vitamin E is necessary for proper foetal and early childhood development [128]; a dietary intake of 12 mg per day during pregnancy is recommended [129]. Supplementation with vitamin E and other micronutrients contributes to the prevention of neural tube defects, and a relationship between plasma levels and cognitive function has been observed [130,131]. Vitamin E has also been linked to problems in pregnancy involving oxidative damage, such as preeclampsia. Anyway, there is no evidence to recommend universal vitamin E supplementation to reduce the risk of maternal–foetal complications [132]. 

Vitamin E deficiency after BS is rare and occurs mainly in malabsorptive diseases, and it takes several years to develop clinical manifestations [133]. Probably for this reason, publications on clinical vitamin E deficiency after bariatric surgery are limited [134]. The ASMBS recommends preventive supplementation in all patients after BS at a dose of 15 mg/day [21,23]. A higher dose is usually needed in BPD. Plasma tocopherol levels are dependent on circulating lipids, and an adjustment for total lipids or plasma cholesterol is recommended [128]. In a study published in Spain, 8.7% and 21.4% of patients with RYGB and BPD, respectively, were found to have vitamin E/cholesterol levels lower than 5 mg/d [135].

Data on the nutritional status of vitamin E in pregnancy after BS are very limited. So far, only one study has been published examining this issue. In a prospective multicentre study of 49 patients, 2% were found to have serum levels below 500 μg/dL in the first trimester. No cases were observed in the second and third trimester [67]. A decrease (below the 2.5th percentile) of vitamin E in cord blood has been observed in cord blood in pregnancy after RYGB compared to a control group (16% vs. 3%) [80].

### 3.7. Vitamin K

Vitamin K is involved in the synthesis of clotting factors and is mainly present in animal fats and oils [136]. It also plays an important role in bone health [137]. Vitamin K deficiency during pregnancy has been related to a higher risk of periventricular and intraventricular haemorrhage, especially in mothers on treatment with anti-epileptic drugs or with malabsorptive conditions [138].

Decreased vitamin K levels have been reported after BS, especially after malabsorptive techniques, and are usually asymptomatic [139]. Data on the nutritional status of vitamin K throughout gestation in women with previous BS are scarce. Some cases of neonatal intracranial haemorrhage have been reported, probably secondary to vitamin K deficiency [140], even after restrictive techniques [141]. A prospective study of 49 post-BS gestational patients and 27 controls found that plasma vitamin K levels were decreased in the first trimester (<0.8 nmol/L) in both groups and were significantly lower in the BS group. Prothrombin time was normal in both groups, although significantly longer in the BS group. Coagulation factors were normal [142].

### 3.8. Iron

Iron is involved in the structure and function of haemoglobin, myoglobin, and enzymes of the respiratory chain. It is absorbed in the duodenum and early jejunal tract and requires an acidic gastric pH [143]. It is found in food in two different forms: as part of the heme group (meat and meat products) and in the non-heme form (legumes, nuts, vegetables), with the former being much more bioavailable. Iron deficiency is very common and causes anaemia, alterations in the mucous membranes, and asthenia, among other symptoms [144]. Obesity itself alters the iron nutritional status, as it constitutes a state of low-grade inflammation, which increases the synthesis of acute-phase reactants, including hepcidin, which decreases iron absorption and also decreases the mobilisation of iron from endogenous stores [145]. Iron intake recommendations depend on its bioavailability in food; EFSA recommends 16 mg/day for women of childbearing age [50].

Iron-deficiency anaemia is one of the most common complications of pregnancy and is a public health problem in many countries. Iron requirements increase during pregnancy because of the increase in total red blood cell volume, up to 20–25% by the end of pregnancy. It is estimated that up to 1200 mg of additional iron is used during pregnancy [146]. Although intestinal absorption also increases, iron intake from food does not appear to be sufficient to maintain adequate iron nutritional status throughout pregnancy, especially if there is a pre-pregnancy iron deficiency state [146]. Anaemia in pregnancy increases maternal morbidity and mortality, hinders foetal growth and maturation, and is associated with an increased risk of low birth weight and alterations in neurocognitive development. In the long term, it promotes obesity and metabolic problems in offspring, including increased vascular risk [146]. The diagnosis of anaemia is established if plasma haemoglobin is less than 11 g/dL in the first and third trimesters and 10.5 g/dL in the second trimester [147]. Iron deficiency during pregnancy is mainly diagnosed based on the determination of plasma ferritin: in general, a cut-off point of 30 μg/L is established [148]. The WHO recommends supplementation with 30–60 mg of elemental iron in all pregnancies [149].

Iron-deficiency anaemia is one of the most frequent nutritional complications of bariatric surgery due to reduced intake and malabsorption, mainly secondary to the modification of gastric pH or the exclusion of the duodenum and proximal jejunum, in addition to possible digestive or menstrual losses [150,151]. The prevalence of iron deficiency and iron-deficiency anaemia ranges from 20 to 70% after RYGB and from 10 to 50% after SG and can require parenteral administration in 2–10% [152]. Preventive supplementation is recommended in techniques that exclude the duodenum at a dose of 40–60 mg/day, especially in high-risk cases (after surgery, women of childbearing age, pregnancy, etc.) [75]. The iron content of conventional multivitamins is not sufficient.

Several studies have found that a history of bariatric surgery increases the risk of iron-deficiency anaemia during pregnancy and that this complication is more common with a longer time after surgery [54,88,153,154]. The prevalence of anaemia ranges from 17 to 70%, with 10–16% requiring intravenous iron and 3–17% requiring transfusion [54]. There is no agreement on the most appropriate pattern of iron supplementation in pregnancy after BS. The recommended dosage ranges from 40 to 600 mg/day, according to different authors [76,154,155,156], but this recommendation is not based on studies specifically designed to evaluate this issue. It should be noted that intravenous iron administration is not indicated during the first trimester of pregnancy [151]. Iron-deficiency anaemia prevention during pregnancy in women with previous BS needs a close follow-up, which should start before conception. The multivitamins designed for pregnancy do not include enough iron for pregnancy after BS. 

### 3.9. Magnesium

Magnesium is an intracellular cation that is part of the bone structure, contributing to proper mineralisation, and is involved as a cofactor of numerous enzymes in muscle contraction, gland secretion, and nerve transmission [157]. Approximately 45% is absorbed in the small intestine via a paracellular diffusion mechanism; a smaller fraction is absorbed in the ileum via transporters (TRPM6 and TRPM7). Magnesium absorption is stimulated by vitamin D and PTH. The plasma Mg concentration is primarily regulated by renal elimination, where approximately 70% of filtered magnesium is reabsorbed [158]. The main sources of magnesium are cereals, nuts, and dairy products. The most characteristic symptoms of magnesium deficiency are anorexia, muscle cramps, rhabdomyolysis, hyperreflexia, convulsions, confusional syndrome, and paralytic ileus. It is not easy to assess the nutritional status of Mg, as it is mainly an intracellular element, and plasma levels may remain within normal limits even when deficiency is present [159]. For this reason, some authors propose raising the reference value for plasma Mg to levels above 2 mg/dL [159].

Magnesium is an important element for pregnancy [160]. Magnesium sulphate is used in the treatment of patients with preeclampsia [161]; it can induce placental vasodilatation, decreases umbilical artery tone, attenuates the effect of endothelin I and angiotensin II on placental vascularisation, and decreases IL-β secretion in placental tissue [162,163]. Plasma Mg levels decrease progressively throughout gestation; this decrease is greater in women with preterm labour [164]. However, no decrease in plasma magnesium has been observed in patients with preeclampsia [164]. The effect of magnesium supplementation on the course and complications of pregnancy is controversial, and the effect probably depends on patient characteristics and maternal magnesium nutritional status [165,166]. There is no evidence to recommend magnesium supplementation to prevent the risk of preeclampsia or other pregnancy complications in healthy women [94,166]. Magnesium intake recommendations do not increase in pregnancy.

Bariatric surgery increases the risk of magnesium deficiency because of decreased intake and malabsorption. However, and probably due to the difficulty in diagnosis, information on magnesium nutritional status after BS is very scarce [76]. There are few data on magnesium deficiency in pregnancy after BS, and, in general, no decrease in serum levels has been observed [93,167].

Considering the difficulty in assessing the nutritional status of magnesium, its importance in gestation, the deficit in magnesium intake in our environment and the possible effect of BS, this is an issue that should be given greater attention in the future. Most multivitamins include a magnesium dosage lower than the recommendations.

### 3.10. Zinc

Zinc is an element involved in the function of more than 200 enzymes, including carbonic anhydrase, DNA polymerase and RNA polymerase. It is directly involved in replication and transcription and plays important roles in growth, foetal nervous system development, and immune response. Zinc is present in foods of animal origin and in cereals and legumes. The recommended intake of zinc is 8–12 mg/day for males and females, respectively [168]. Zinc deficiency is very common in developing countries and has very important clinical consequences: retarded growth and sexual maturation, asthenia, dermatitis, hypogonadism, altered sense of taste, and other general manifestations [169]. Zinc deficiency is generally related to decreased intake or availability. It has also been described in numerous clinical conditions associated with malabsorption, such as short bowel syndrome, inflammatory bowel disease, and coeliac disease [170].

Zinc is directly involved in foetal growth and development, especially in the nervous system, and in the immune response [171]. There is active transport in the placenta, so levels in the cord blood are higher than in the mother’s blood [172]. In animals, zinc deficiency leads to an increased risk of miscarriage, placental abnormalities, congenital malformations, and intrauterine growth retardation [171,173]. In humans, decreased plasma zinc levels have been associated with decreased birth weight and preeclampsia in some studies, but not in others [174,175]. In a recently published meta-analysis, a significant pooled correlation was found between umbilical cord blood zinc concentrations and birth weight (r: 0.09, 95% CI: 0.04 to 0.15) [176].

Zinc deficiency is common in BS patients, even before surgery [21], especially in mixed or malabsorptive techniques [88]. Most cases are asymptomatic, although low intake and Zn deficiency have been linked to alopecia after BS [177]. The ASMBS, recommends assessing nutritional status prior to BS, keeping in mind that plasma levels may fall in relation to obesity itself. In addition, preventive supplementation is recommended in all patients, with a dose that depends on the surgical technique used: malabsorptive techniques: 16–22 mg/day; gastric bypass: 8–22 mg/day; and restrictive techniques: 8–11 mg/day [21,23].

Data on the nutritional status of zinc and its clinical consequences for pregnancy after bariatric surgery are limited. In a study of 30 patients with a history of RYGB, 20% showed decreased plasma levels in the first and third trimesters, with no relation to maternal anthropometry or newborn weight [178]. Another study, conducted in 56 patients, describes plasma zinc levels of 13.1 ± 2.6, 11.5 ± 1.7, and 10.7 ± 1.4 μmol.

L in the three trimesters, respectively. They found no cases of deficiency, no differences between the different techniques, and no effects on maternal and foetal outcomes [93]. In a prospective study in 87 women [179], zinc deficiency (<0.51 mg/L) was found in 8.0%, all after RYGB (18.9% vs. 0% in SG; *p* = 0.02), and preterm birth occurred in 100% of these cases. The usual multivitamins do not provide sufficient zinc for pregnancy after bariatric surgery, especially in the case of malabsorptive techniques. It is also necessary to consider that the pharmacological use of some nutrients, such as iron, decreases the bioavailability of zinc from food and that a high dose of zinc decreases the absorption of copper. 

### 3.11. Copper

Copper is an essential element that acts as a ligand for numerous proteins and enzymes (superoxide dismutase, ferroxidase, amino oxidase, cytochrome-C oxidase, etc.). It is involved in antioxidant protection, in the transport of iron and other metals, and in the metabolism of amino acids. It is absorbed in the stomach and duodenum, and gastric acid contributes to the release of Cu from food. Copper requirements according to the RDA are 900 µg/day in adults. In pregnant and breastfeeding women, the requirements increase, and more than 1000 µg/day is necessary [168]. Copper is an essential element in brain development [180] and has been linked to neurodegenerative diseases [181]. Copper deficiency leads to a clinical picture with haematological (anaemia, leukopenia, or pancytopenia) and neurological (myelopathy and peripheral neuropathy) manifestations [182].

Copper deficiency can have negative effects on embryonic and foetal development [183]. Copper is involved in the normal functioning of numerous enzymes, so its deficiency alters ATP production, lipid peroxidation, hormone activation, and angiogenesis and causes pulmonary and skeletal alterations. Genetic alterations in copper metabolism lead to alterations in embryonic and foetal development that may increase mortality. Menken’s disease is transmitted in an X-linked recessive manner and usually results in the death of the child before the age of 5 years after presenting with clinical signs of neuronal and connective tissue degeneration. Studies have shown that, in normal gestations, copper levels increase progressively throughout pregnancy [184] due to an increase in ceruloplasmin secondary to gestational hyperestrogenism and a decrease in biliary copper excretion [185]. Maternal copper deficiency increases the risk of prematurity and low-birth-weight infants, and a decrease in cord blood copper has been observed in low-birth-weight infants [183]. Copper deficiency during pregnancy has also been associated with an increased risk of the premature rupture of membranes and preterm birth [186].

Copper deficiency is rare in the Western population but has been increasing in recent years as a consequence of drug treatment and especially in relation to bariatric surgery [182,187]. Its prevalence is estimated at 9.6% after RYGB [188]. Copper deficiency is a recognised cause of neurological impairment after BS, especially with techniques that exclude the duodenum, the main site of absorption [189]. Several studies have observed a decrease in plasma copper levels after bariatric bypass surgery [190]. Clinical copper deficiency is less common but can be severe; several cases have been reported, mainly after malabsorptive techniques, with neurological impairment and/or anaemia [191,192]. It is recommended that all patients receive copper supplementation, at a dose depending on the surgical technique used [21,23]: in patients with SG or GB, 1 mg/day, and in patients with RYGB or BPD, 2 mg/day, in the form of copper sulphate or gluconate. Zinc treatment may decrease copper absorption [193]. Cu supplementation of 1–2 mg for every 8–15 mg of zinc is recommended [21].

There are no data on plasma copper levels in pregnancy after BS or the possible impact on the course and complications of pregnancy. No cases of clinical copper deficiency during pregnancy after BS have been reported. Decreased plasma copper levels in pregnant women with a history of bariatric surgery could have adverse consequences on foetal development or increase the risk of complications. Although there are no specific recommendations on Cu supplementation in pregnancy after BS, these women should receive at least similar supplementation to that recommended in BS in general [75,76]. This dose is safe in pregnancy and does not reach the maximum tolerable intake (10 mg/day) [182]. It should be noted that most multivitamins designed for pregnancy do not provide copper. 

### 3.12. Selenium

Selenium plays an important role in maintaining redox balance through selenoproteins such as glutathione peroxidase (GSH) [194]. Plasma values above 70 μg/L optimise GSH function [195]. It is absorbed in the upper sections of the small intestine, mainly in the duodenum. Se intake and nutritional status are highly dependent on the geographical area. The EFSA recommends an intake of selenium in the adult population of 70 μg/day for men and women, and a similar amount is recommended during pregnancy. During breastfeeding, on the other hand, considering the Se content in breast milk, an intake of 85 μg/day is recommended, similar to that for non-pregnant women [196].

A decrease in selenium levels and GSH peroxidase activity has been observed in pregnancy [107] and has been linked to some complications, such as preeclampsia [197,198]. In a recently published systematic review that included 26 studies with 1855 preeclampsia cases compared with 3728 healthy pregnant controls, the level of selenium was significantly lower in cases of preeclampsia compared with the controls (SMD = −0.85; 95% confidence interval: −1.46, −0.25; *p* < 0.01). A decrease in serum Se has been observed throughout gestation [199], with no association with the risk of SGA [200].

There are few data on the nutritional status of selenium in relation to bariatric surgery. Several cases of cardiomyopathy secondary to selenium deficiency after bariatric surgery have been reported [201,202], which have improved after specific supplementation. In patients who are candidates for BS, decreased selenium serum levels have been observed compared to a control group [203]. A decrease has also been described after BS [204], which can be prevented by micronutrient supplementation [205]. Although Se deficiency may be more frequent in techniques that exclude the duodenum, decreased levels have also been reported after sleeve gastrectomy [206]. In a systematic review and meta-analysis that included nine studies with a total of 1174 patients, selenium deficiency prevalence was 16% and 2% at 1- and 2-year follow-ups after BS, respectively [207]. Symptoms included weakness, myopathy and cardiomyopathy, loss of muscle mass, erythematous desquamating eruption, lethargy, dyspnoea, and bilateral lower extremity pitting oedema.

The prevalence of selenium deficiency in pregnancy after BS was evaluated in a retrospective study including 57 singleton pregnancies [93]. Selenium serum levels were low in 77.8%, 22.2%, and 50.0% in the first, second, and third trimesters, respectively. In a prospective study in 87 women [179], selenium deficiency (<60 μg/L) was found in 17.2%: 21.6% after RYGB and 14.0% after SG (*p* = 0.36). A selenium deficit in the second trimester in women with a history of BS was negatively correlated with birthweight and with birthweight z-score [179].

### 3.13. Iodine

Iodine is an essential element whose main function is its participation in the synthesis of thyroid hormones. It is a critical nutrient in cellular metabolism and in the development of the nervous system in the prenatal and postnatal periods [208]. The main sources of iodine are dairy products, fish, eggs, and, above all, fortified foods such as iodised salt. Iodine deficiency is a major public health problem and one of the preventable causes of stunted growth and impaired neurological development [208].

Iodine requirements increase during pregnancy and lactation, and an intake of 200–250 μg/day is recommended. All women of childbearing age should ensure an adequate iodine intake at least one year before pregnancy. The intake of iodine in food and iodised salt is generally sufficient to meet these requirements. However, considering the risk of iodine deficiency for foetal development, iodine supplementation should be recommended in cases where there is a risk of insufficient intake [209].

Very few studies have been published on the impact of barbaric surgery on iodine absorption or metabolism. So far, three studies have been published. Urinary iodine excretion had not changed 6 months after malabsorptive BS, nor had autoimmunity or thyroid gland volume [210]. Another study compared the nutritional status of iodine in three groups of women: morbidly obese, patients after bariatric surgery with at least 18 months of follow-up after bariatric surgery, and a normal-weight control group. Obese women had a significantly lower urinary iodine concentration (UIC, μg/g creatinine) in comparison with non-obese women (96.6 [25.8–267.3] vs. 173.3 [47.0–493.6] μg/g; *p* < 0.001), with a lower proportion of subjects with an adequate iodine status (46.6 vs. 83.3%, *p* < 0.001). Mean UIC was higher in women with previous BS in comparison with women with obesity (131.9 [62.9–496.4] vs. 96.6 [25.8–267.3] μg/g; *p* < 0.001). No difference in UIC was found between RYGB and SG. UIC was negatively correlated with BMI (r = −0.278, *p* < 0.001). Multiple linear regression analyses showed that BMI was independently associated with UIC (beta = −0.312, *p* < 0.001; R (2) = 0.166). In this study, women consuming iodised salt were excluded [211]. The SOS study investigators evaluated 188 patients after RYGB and 188 after SG, at least 10 years after BS, compared with a control group, and did not observe a higher prevalence of iodine deficiency [212]. BS has not been shown to impair iodine absorption or metabolism or to induce iodine deficiency, so specific supplementation recommended after bariatric surgery does not need to include iodine in iodine-sufficient countries, and ensuring that the dietary intake of iodine in food and iodised salt is sufficient.

No studies have been published evaluating the iodine nutritional status during pregnancy in women with previous BS. In agreement with previously described studies, in these women, the same recommendation of iodine intake can be made as in the general pregnant population, 200–250 μg/day.

There is no agreement on the most appropriate preventive micronutrient supplementation during gestation in women with previous bariatric surgery, and, due to the paucity of data, recommended doses are generally based on expert opinion. Table 5 includes a summary of these recommendations [31,32,33,154,213].

## 4. Conclusions

Obesity increases the risk of complications during gestation, such as gestational diabetes, preeclampsia, or macrosomia. Bariatric surgery may reduce these risks but may induce mineral and micronutrient deficiencies, which can have adverse consequences for the short- and long-term health of the mother and her offspring. This narrative review of the literature enables us to offer some recommendations to optimise the follow-up of pregnancy in women with previous bariatric surgery [28,29,30,31,32,33,34] (Table 6). Further studies are needed to identify the factors that promote micronutrient deficiencies during pregnancy after bariatric surgery, the effects on maternal and foetal health and long-term outcomes, and the most effective preventive treatment.

## Figures and Tables

**Table 1:** Clinical consequences of maternal obesity and excess weight gain in pregnancy.

Clinical Consequences of Maternal Obesity and Excess Weight Gain in Pregnancy	
Maternal	
Pre-conception	Higher risk of type 2 diabetes, high blood pressure, and infertility.
Pregnancy	Previous metabolic diseases, gestational diabetes, hypertension, deep vein thrombosis, pulmonary thromboembolism, depression.
Delivery	Higher risk of complications, instrumental delivery, caesarean, higher anaesthetic risk.
Postpartum	Infection, depression, failure in breastfeeding, weight retention, obesity
**Newborn and infant**	
	Macrosomia, large-for-gestational-age newborn, prematurity, shoulder dystocia, birth defects, neonatal hypoglycaemia.
**Long term**	
	Higher risk of obesity, metabolic complications. Higher vascular risk for both the mother and offspring.

**Table 2:** Main micronutrient deficiencies after bariatric surgery.

Surgical Technique	Physiopathological Factors	Most Affected Micronutrients
Gastric banding	Decrease intake Food intolerance (meat, milk)	All micronutrients, especially those with low body stores (thiamine) Iron, zinc, calcium, vit D
Sleeve gastrectomy	Decrease intake Food intolerance (meat, milk) Gastrectomy	All micronutrients, especially those with low body stores (thiamine) Iron, zinc, calcium, vit D Vitamin B12, iron, calcium
Roux-Y gastric bypass	Decrease intake Food intolerance (meat, milk) Gastrectomy Duodenal/jejunal exclusion	All micronutrients, especially those with low body stores (thiamine) Iron, zinc, calcium, vit D Vitamin B12, iron, calcium Iron, calcium, zinc, copper, liposoluble vitamins
Biliopancreatic diversion duodenal switch SADI-S	Decrease intake Food intolerance (meat, milk) Gastrectomy Duodenal/jejunal exclusion Steatorrhea	All micronutrients, especially those with low body stores (thiamine) Iron, zinc, calcium, vit D Vitamin B12, iron, calcium Iron, calcium, zinc, copper, liposoluble vitamins Calcium, liposoluble vitamins

**Table 3:** Proposal of micronutrient recommendations in pregnancy after bariatric surgery.

Micronutrient	SG/RYGB	BPD and Other Malabsorptive Procedures
Folate	400–8008 μg/d 800–1000 μg/d in women of childbearing age	
Vitamin B12	350–1000 μg/d (oral or sublingual) 1000 μg/month IM-SC)	
Thiamine	12 mg/d Increase to 100–300 mg if low intake, nausea/vomiting	
Vitamin D	3000 UI/d (Vit D > 30 ng/mL)	A higher dose is usually needed
Vitamin A	800–3000 μg/d	3000 μg/d (10.000 UI)
Vitamin E	15 mg/d	90 mg/d
Vitamin K	50–120 μg/d	300 μg/d
Iron	SG: male or non-menstruating: 18 mg/d Menstruating female or RYGB/BPD: 45–60 mg/d	
Calcium	1200–1500 mg/d	1800–2400 mg/d
Magnesium	350 mg/d	350 mg/d
Zinc	SG: 8–11 mg/d RYGB: 8–22 mg/d	16–22 mg/d
Copper	SG: 1 mg/d RYGB 2 mg/d	2 mg/d

**Table 4:** Clinical consequences of micronutrient deficiency during pregnancy.

Micronutrient	Clinical Consequences of Deficiency for Maternal–Foetal Health
Folate	Neural tube defects, miscarriage, abruptio placentae, prematurity
Vitamin B12	Abortion, prematurity, growth retardation, neural tube defects, cognitive impairment
Thiamine	Risk of thiamine deficiency in hyperemesis gravidarum
Vitamin D	Gestational diabetes, preeclampsia, low birth weight, long-term complications
Vitamin A	Foetal malformations, pulmonary dysplasia, anaemia
Vitamin E	Preeclampsia, neural tube defects, cognitive impairment, haemolytic disease of the newborn
Vitamin K	Periventricular and intraventricular haemorrhage
Iron	Increased maternal and foetal morbidity and mortality, miscarriage, decreased weight and foetal development
Zinc	Delayed foetal growth and maturation, prematurity
Copper	Abortion, prematurity, low weight
Selenium	Preeclampsia
Iodine	Alteration in the development of central nervous system, mental retardation

**Table 5:** Summary of micronutrient recommendations in pregnancy after bariatric surgery.

Micronutrient	Dose	Comment
Folate	800–1000 μg	Higher dose in women with obesity (1–5 mg)
Vitamin B12	1000–2000 μg/1–3 months, i.m.	Can also be administered orally (>350–500 μg/d)
Thiamine	12 mg	Increase to 100–300 mg if low intake, nausea/vomiting
Vitamin D	2000–4000 UI	Vitamin D > 30 ng/mL
Vitamin A	800–1500 μg	A dose below 3000 μg (10.000 UI) is safe in pregnancy; the retinol form of vitamin A should be avoided
Vitamin E	15 mg	Monitoring in malabsorptive techniques
Vitamin K	50–120 μg/d	Higher risk of deficiency in premature newborns
Iron	100–200 mg	Ferritin > 30 mg/L, gradual increase of dose; i.v. iron is not recommended in first term
Calcium	1500–2400 mg	Increase dietary intake; separated from iron supplement
Magnesium	350 mg	Multivitamins usually contain a lower dose
Zinc	12–30 mg	Can decrease copper absorption
Copper	1–2 mg	Separated from zinc supplements
Selenium	50–60 μg	Monitor in malabsorptive techniques
Iodine	200–250 μg	The same dose as that in normal pregnancy

**Table 6:** Pregnancy after bariatric surgery: summary of recommendations.

Pregnancy in Women with Previous Bariatric Surgery: Summary of Recommendations
Appropriate selection of the bariatric surgical technique. Non-malabsorptive techniques should, in general, be preferred.
Appropriate follow-up after bariatric surgery, with the necessary supplementation to prevent and treat possible nutritional deficiencies.
Preferably, the onset of pregnancy should be delayed by 12–18 months after bariatric surgery. Pre-conceptional clinical and nutritional assessment is recommended.
Follow-up during pregnancy should be carried out by a multidisciplinary team.
Close monitoring of the patient if oral tolerance is inadequate or vomiting occurs. It is advisable to increase the thiamine dose to 100–300 mg/day.
Monitoring of maternal weight gain and intrauterine growth. Consider oral nutritional supplements and/or pancreatic enzymes.
Preventive supplementation with minerals and micronutrients, at the necessary dose, depending on the type of bariatric surgery and clinical and analytical evolution.
Iodine recommendations are similar to those for women who have not undergone bariatric surgery.
Screening for gestational complications, following specific protocols. In the case of gestational diabetes, it is recommended to avoid oral glucose overload.
Monitoring for the occurrence of surgical complications, such as internal hernia, a serious but rare clinical condition that requires a specific diagnostic approach and treatment.
Encourage lactation with a close clinical and nutritional follow-up.

## Data Availability

Not applicable.

## References

[1] Mechanick J.I., Hurley D.L., Garvey W.T. (2016). Adiposity-Based Chronic Disease as a new Diagnostic Term: The American Association of Clinical Endocrinologists and American College of Endocrinology Position Statement. Endocr. Pract..

[2] Garvey W.T., Mechanick J.I., Brett E.M., Garber A.J., Hurley D.L., Jastreboff A.M., Nadolsky K., Pessah-Pollack R., Plodkowski R., Reviewers of the AACE/ACE Obesity Clinical Practice Guidelines (2016). American association of clinical endocrinologists and american college of endocrinology comprehensive clinical practice guidelines for medical care of patients with obesity—Executive Summary. Endocr. Pract..

[3] Rittenberg V., Seshadri S., Sunkara S.K., Sobaleva S., Oteng-Ntim E., El-Toukhy T. (2011). Effect of body mass index on IVF treatment outcome: An updated systematic review and meta-analysis. Reprod. Biomed. Online.

[4] Bellver J., Busso C., Pellicer A., Remohí J., Simón C. (2006). Obesity and assisted reproductive technology outcomes. Reprod. Biomed. Online.

[5] Yu Z., Han S., Zhu J., Sun X., Ji C., Guo X. (2013). Pre-Pregnancy Body Mass Index in Relation to Infant Birth Weight and Offspring Overweight/Obesity: A Systematic Review and Meta-Analysis. PLoS ONE.

[6] Marchi J., Berg M., Dencker A., Olander E.K., Begley C. (2015). Risks associated with obesity in pregnancy, for the mother and baby: A systematic review of reviews. Obes. Rev..

[7] Goldstein R.F., Abell S.K., Ranasinha S., Misso M., Boyle J.A., Black M.H., Li N., Hu G., Corrado F., Rode L. (2017). Association of Gestational Weight Gain with Maternal and Infant Outcomes: A Systematic Review and Meta-analysis. JAMA.

[8] Santos S., Voerman E., Amiano P., Barros H., Beilin L., Bergström A., Charles M., Chatzi L., Chevrier C., Chrousos G. (2019). Impact of maternal body mass index and gestational weight gain on pregnancy complications: An individual participant data meta-analysis of European, North American, and Australian cohorts. BJOG.

[9] Martin A., Krishna I., Ellis J., Paccione R., Badell M. (2014). Super obesity in pregnancy: Difficulties in clinical management. J. Perinatol..

[10] Crane J.M., Murphy P., Burrage L., Hutchens D. (2013). Maternal and Perinatal Outcomes of Extreme Obesity in Pregnancy. J. Obstet. Gynaecol. Can..

[11] Arterburn D.E., Courcoulas A.P. (2014). Bariatric surgery for obesity and metabolic conditions in adults. BMJ.

[12] Angrisani L., Santonicola A., Iovino P., Ramos A., Shikora S., Kow L. (2021). Bariatric Surgery Survey 2018: Similarities and Disparities Among the 5 IFSO Chapters. Obes. Surg..

[13] Batterham R.L., Cummings D.E. (2016). Mechanisms of Diabetes Improvement Following Bariatric/Metabolic Surgery. Diabetes Care.

[14] Hutch C.R., Sandoval D.A. (2017). Physiological and molecular responses to bariatric surgery: Markers or mechanisms underlying T2DM resolution?. Ann. N. Y. Acad. Sci..

[15] Davies N.K., O’Sullivan J.M., Plank L.D., Murphy R. (2019). Altered gut microbiome after bariatric surgery and its association with metabolic benefits: A systematic review. Surg. Obes. Relat. Dis..

[16] Penney N.C., Kinross J., Newton R.C., Purkayastha S. (2015). The role of bile acids in reducing the metabolic complications of obesity after bariatric surgery: A systematic review. Int. J. Obes..

[17] Vítek L., Haluzík M. (2016). The role of bile acids in metabolic regulation. J. Endocrinol..

[18] Syn N.L., E Cummings D., Wang L.Z., Lin D.J., Zhao J.J., Loh M., Koh Z.J., Chew C.A., Loo Y.E., Tai B.C. (2021). Association of metabolic–bariatric surgery with long-term survival in adults with and without diabetes: A one-stage meta-analysis of matched cohort and prospective controlled studies with 174 772 participants. Lancet.

[19] Wiggins T., Guidozzi N., Welbourn R., Ahmed A.R., Markar S.R. (2020). Association of bariatric surgery with all-cause mortality and incidence of obesity-related disease at a population level: A systematic review and meta-analysis. PLOS Med..

[20] Amaya García M.J., Vilchez López F.J., Campos Martín C., Sánchez Vera P., Pereira Cunill J.L. (2012). Micronutrientes en Cirugía Bariátrica. Nutr. Hosp..

[21] Parrott J., Frank L., Rabena R., Craggs-Dino L., Isom K.A., Greiman L. (2019). American Society for Metabolic and Bariatric Surgery Integrated Health Nutritional Guidelines for the Surgical Weight Loss Patient 2016 Update: Micronutrients. Surg. Obes. Relat. Dis..

[22] Jensterle M., Rizzo M., Janez A. (2021). Glucagon-Like Peptide 1 and Taste Perception: From Molecular Mechanisms to Potential Clinical Implications. Int. J. Mol. Sci..

[23] Mechanick J.I., Apovian C., Brethauer S., Garvey W.T., Joffe A.M., Kim J., Kushner R.F., Lindquist R., Pessah-Pollack R., Seger J. (2020). Clinical practice guidelines for the perioperative nutrition, metabolic, and nonsurgical support of patients undergoing bariatric procedures—2019 update: Cosponsored by American Association of Clinical Endocrinologists/American College of Endocrinology, The Obesity Society, American Society for Metabolic & Bariatric Surgery, Obesity Medicine Association, and American Society of Anesthesiologists. Surg. Obes. Relat. Dis..

[24] O’Kane M., Parretti H.M., Pinkney J., Welbourn R., Hughes C.A., Mok J., Walker N., Thomas D., Devin J., Coulman K.D. (2020). British Obesity and Metabolic Surgery Society Guidelines on perioperative and postoperative biochemical monitoring and micronutrient replacement for patients undergoing bariatric surgery—2020 update. Obes. Rev..

[25] Galazis N., Docheva N., Simillis C., Nicolaides K.H. (2014). Maternal and neonatal outcomes in women undergoing bariatric surgery: A systematic review and meta-analysis. Eur. J. Obstet. Gynecol. Reprod. Biol..

[26] Yi X.Y., Li Q.F., Zhang J., Wang Z.H. (2015). A meta-analysis of maternal and fetal outcomes of pregnancy after bariatric surgery. Int. J. Gynaecol. Obstet..

[27] Kwong W., Tomlinson G., Feig D.S. (2018). Maternal and neonatal outcomes after bariatric surgery; a systematic review and meta-analysis: Do the benefits outweigh the risks?. Am. J. Obstet. Gynecol..

[28] Badreldin N., Kuller J., Rhee E., Brown L., Laifer S. (2016). Pregnancy Management After Bariatric Surgery. Obstet. Gynecol. Surv..

[29] Harreiter J., Schindler K., Bancher-Todesca D., Göbl C., Langer F., Prager G., Gessl A., Leutner M., Ludvik B., Luger A. (2018). Management of Pregnant Women after Bariatric Surgery. J. Obes..

[30] González I., Lecube A., Rubio M., García-Luna P.P. (2016). Pregnancy after bariatric surgery: Improving outcomes for mother and child. Int. J. Women’s Health.

[31] Shawe J., Ceulemans D., Akhter Z., Neff K., Hart K., Heslehurst N., Štotl I., Agrawal S., Steegers-Theunissen R., Taheri S. (2019). Pregnancy after bariatric surgery: Consensus recommendations for periconception, antenatal and postnatal care. Obes. Rev..

[32] Alamri S.H., Abdeen G.N. (2022). Maternal Nutritional Status and Pregnancy Outcomes Post-bariatric Surgery. Obes. Surg..

[33] Huang B., Yo J.H., Gandhi S., Maxwell C. (2022). Micronutrient screening, monitoring, and supplementation in pregnancy after bariatric surgery. Obstet. Med..

[34] Guthrie T.M., Dix C.F., Truby H., Kumar S., de Jersey S.J. (2023). A Systematic Review Investigating Maternal Nutrition During Pregnancy After Bariatric Surgery. Obes. Surg..

[35] Akhter Z., Rankin J., Ceulemans D., Ngongalah L., Ackroyd R., Devlieger R., Vieiram R., Heslehurst N. (2018). Investigating the association between pregnancy following bariatric surgery and adverse perinatal outcomes: A systematic review and meta-analysis. PLoS Med..

[36] Cooper N., Fiennes A., Hashemi M., Adamo M., El-Kalaawy M. (2010). Pregnancy in first 12 months after bariatric surgery. Surg. Obes. Relat. Dis..

[37] Johansson K., Cnattingius S., Näslund I., Roos N., Lagerros Y.T., Granath F., Stephansson O., Neovius M. (2015). Outcomes of Pregnancy after Bariatric Surgery. N. Engl. J. Med..

[38] Heusschen L., Krabbendam I., van der Velde J.M., Deden L.N., Aarts E.O., Merién A.E.R., Emous M., Bleumink G.S., Lutgers H.L., Hazebroek E.J. (2021). A Matter of Timing—Pregnancy After Bariatric Surgery. Obes. Surg..

[39] Harrison R.K., Berkelhammer C., Suarez V., Kay H.H. (2018). Managing Malnourishment in Pregnancy after Bariatric Surgery. J. Obstet. Gynaecol. Can..

[40] Karmon A., Sheiner E. (2008). Pregnancy after bariatric surgery: A comprehensive review. Arch. Gynecol. Obstet..

[41] Beiglböck H., Mörth E., Reichardt B., Stamm T., Itariu B., Harreiter J., Eichelter J., Prager G., Kautzky-Willer A., Wolf P. (2023). The Timing of Pregnancies After Bariatric Surgery has No Impact on Children’s Health—A Nationwide Population-based Registry Analysis. Obes. Surg..

[42] Mensink G.B.M., Fletcher R., Gurinović M., Huybrechts I., Lafay L., Serra-Majem L., Szponar L., Tetens I., Verkaik-Kloosterman J., Baka A. (2013). Mapping low intake of micronutrients across Europe. Br. J. Nutr..

[43] Das J.K., Salam R.A., Kumar R., Bhutta Z.A. (2013). Micronutrient fortification of food and its impact on woman and child health: A systematic review. Syst. Rev..

[44] Maslin K., James A., Brown A., Bogaerts A., Shawe J. (2019). What Is Known About the Nutritional Intake of Women during Pregnancy Following Bariatric Surgery? A Scoping Review. Nutrients.

[45] Lecube A., de Hollanda A., Calañas A., Vilarrasa N., Rubio M.A., Breton I., Goday A., Vidal J., Iglesias P., Fernández-Soto M.L. (2015). Trends in Bariatric Surgery in Spain in the Twenty-First Century: Baseline Results and 1-Month Follow Up of the RICIBA, a National Registry. Obes. Surg..

[46] Alanis M.C., Goodnight W.H., Hill E.G., Robinson C.J., Villers M.S., Johnson D.D. (2010). Maternal super-obesity (body mass index ≥ 50) and adverse pregnancy outcomes. Acta Obstet. Gynecol. Scand..

[47] Cogswell M.E., Weisberg P., Spong C. (2003). Cigarette smoking, alcohol use and adverse pregnancy outcomes: Implications for micronutrient supplementation. J. Nutr..

[48] Abbassi-Ghanavati M., Greer L.G., Cunningham F.G. (2009). Pregnancy and Laboratory Studies. Obstet. Gynecol..

[49] Bailey L.B., Stover P.J., McNulty H., Fenech M.F., Gregory J.F., Mills J.L., Pfeiffer C.M., Fazili Z., Zhang M., Ueland P.M. (2015). Biomarkers of Nutrition for Development—Folate Review. J. Nutr..

[50] EFSA Panel on Dietetic Products, Nutrition and Allergies (NDA) (2015). Scientific Opinion on Dietary Reference Values for iron. EFSA J..

[51] MRC Vitamin Study Research Group (1991). Prevention of neural tube defects: Results of the Medical Research Council Vitamin Study. Lancet.

[52] Shaw G.M., Carmichael S.L., Nelson V., Selvin S., Schaffer D.M. (2004). Occurrence of Low Birthweight and Preterm Delivery among California Infants before and after Compulsory Food Fortification with Folic Acid. Public Health Rep..

[53] Rodríguez L., Médico M. (2010). Suplementos en embarazadas: Controversias, evidencias y recomendaciones. Inf. Ter. Sist. Nac. Salud..

[54] Rottenstreich A., Elazary R., Goldenshluger A., Pikarsky A.J., Elchalal U., Ben-Porat T. (2019). Maternal nutritional status and related pregnancy outcomes following bariatric surgery: A systematic review. Surg. Obes. Relat. Dis..

[55] Mojtabai R. (2004). Body mass index and serum folate in childbearing age women. Eur. J. Epidemiol..

[56] De-Regil L.M., Peña-Rosas J.P., Fernández-Gaxiola A.C., Rayco-Solon P. (2015). Effects and safety of periconceptional oral folate supplementation for preventing birth defects. Cochrane Database Syst. Rev..

[57] Lassi Z.S., Salam R.A., Haider B.A., Bhutta Z.A. (2013). Folic acid supplementation during pregnancy for maternal health and pregnancy outcomes. Cochrane Database Syst. Rev..

[58] Moussa H.N., Nasab S.H., A Haidar Z., Blackwell S.C., Sibai B.M., Srikrishnan A., Dash P., Divieto C., Sassi M.P., Abdelhafiz A.H. (2016). Folic acid supplementation: What is new? Fetal, obstetric, long-term benefits and risks. Futur. Sci. OA.

[59] E Brolin R., Gorman R.C., Milgrim L.M., A Kenler H. (1991). Multivitamin prophylaxis in prevention of post-gastric bypass vitamin and mineral deficiencies. Int. J. Obes..

[60] MacLean L.D. (1984). Nutrition following intestinal bypass and gastric operations for morbid obesity. Can. J. Surg..

[61] Gasteyger C., Suter M., Calmes J.M., Gaillard R.C., Giusti V. (2006). Changes in Body Composition, Metabolic Profile and Nutritional Status 24 Months after Gastric Banding. Obes. Surg..

[62] Aasheim E.T., Björkman S., Søvik T.T., Engström M., E Hanvold S., Mala T., Olbers T., Bøhmer T. (2009). Vitamin status after bariatric surgery: A randomized study of gastric bypass and duodenal switch. Am. J. Clin. Nutr..

[63] Skroubis G., Sakellaropoulos G., Pouggouras K., Mead N., Nikiforidis G., Kalfarentzos F. (2002). Comparison of Nutritional Deficiencies after Rouxen-Y Gastric Bypass and after Biliopancreatic Diversion with Roux-en-Y Gastric Bypass. Obes. Surg..

[64] Weng T.-C., Chang C.-H., Dong Y.-H., Chang Y.-C., Chuang L.-M. (2015). Anaemia and related nutrient deficiencies after Roux-en-Y gastric bypass surgery: A systematic review and meta-analysis. BMJ Open.

[65] Moliterno J.A., DiLuna M.L., Sood S., Roberts K.E., Duncan C.C. (2008). Gastric bypass: A risk factor for neural tube defects?. J. Neurosurg. Pediatr..

[66] Pelizzo G., Calcaterra V., Fusillo M., Nakib G., Ierullo A.M., Alfei A., Spinillo A., Stronati M., Cena H. (2014). Malnutrition in pregnancy following bariatric surgery: Three clinical cases of fetal neural defects. Nutr. J..

[67] Devlieger R., Guelinckx I., Jans G., Voets W., Vanholsbeke C., Vansant G. (2014). Micronutrient Levels and Supplement Intake in Pregnancy after Bariatric Surgery: A Prospective Cohort Study. PLoS ONE.

[68] Bebber F.E., Rizzolli J., Casagrande D.S., Rodrigues M.T., Padoin A.V., Mottin C.C., Repetto G. (2011). Pregnancy after Bariatric Surgery: 39 Pregnancies Follow-up in a Multidisciplinary Team. Obes. Surg..

[69] González I., Rubio M.A., Cordido F., Bretón I., Morales M.J., Vilarrasa N., Monereo S., Lecube A., Caixàs A., Vinagre I. (2014). Maternal and Perinatal Outcomes After Bariatric Surgery: A Spanish Multicenter Study. Obes. Surg..

[70] O’leary F., Samman S. (2010). Vitamin B12 in Health and Disease. Nutrients.

[71] Finkelstein J.L., Layden A.J., Stover P.J. (2015). Vitamin B-12 and Perinatal Health. Adv. Nutr. Int. Rev. J..

[72] Rogne T., Tielemans M.J., Chong M.F.-F., Yajnik C.S., Krishnaveni G.V., Poston L., Jaddoe V.W.V., Steegers E.A.P., Joshi S., Chong Y.-S. (2017). Associations of Maternal Vitamin B12 Concentration in Pregnancy with the Risks of Preterm Birth and Low Birth Weight: A Systematic Review and Meta-Analysis of Individual Participant Data. Am. J. Epidemiol..

[73] Majumder S., Soriano J., Cruz A.L., Dasanu C.A. (2013). Vitamin B12 deficiency in patients undergoing bariatric surgery: Preventive strategies and key recommendations. Surg. Obes. Relat. Dis..

[74] Smelt H.J.M., Pouwels S., Smulders J.F. (2017). Different Supplementation Regimes to Treat Perioperative Vitamin B12 Deficiencies in Bariatric Surgery: A Systematic Review. Obes. Surg..

[75] Mechanick J., Youdim A., Jones D., Timothy Garvey W., Hurley D.L., Mc Mahon M. (2013). AACE/TOS/ASMBS Bariatric Surgery Clinical Practice Guidelines. Endocr. Pract..

[76] Busetto L., Dicker D., Azran C., Batterham R.L., Farpour-Lambert N., Fried M., Hjelmesæth J., Kinzl J., Leitner D.R., Makaronidis J.M. (2018). Obesity Management Task Force of the European Association for the Study of Obesity Released “Practical Recommendations for the Post-Bariatric Surgery Medical Management”. Obes. Surg..

[77] Grange D.K., Finlay J.L. (1994). Nutritional Vitamin B_12_ Deficiency in a Breastfed Infant Following Maternal Gastric Bypass. Pediatr. Hematol. Oncol..

[78] Wardinsky T.D., Montes R.G., Friederich R.L., Broadhurst R.B., Sinnhuber V., Bartholomew D. (1995). Vitamin B12 Deficiency Associated With Low Breast-Milk Vitamin B12 Concentration in an Infant Following Maternal Gastric Bypass Surgery. Arch. Pediatr. Adolesc. Med..

[79] Celiker M.Y., Chawla A. (2009). Congenital B12 deficiency following maternal gastric bypass. J. Perinatol..

[80] Gascoin G., Gerard M., Sallé A., Becouarn G., Rouleau S., Sentilhes L., Coutant R. (2017). Risk of low birth weight and micronutrient deficiencies in neonates from mothers after gastric bypass: A case control study. Surg. Obes. Relat. Dis..

[81] Mead N.C., Sakkatos P., Sakellaropoulos G.C., Adonakis G.L., Alexandrides T.K., Kalfarentzos F. (2014). Pregnancy outcomes and nutritional indices after 3 types of bariatric surgery performed at a single institution. Surg. Obes. Relat. Dis..

[82] Sriram K., Manzanares W., Joseph K. (2012). Thiamine in Nutrition Therapy. Nutr. Clin. Pract..

[83] Ashraf V., Prijesh J., Praveenkumar R., Saifudheen K. (2016). Wernicke’s encephalopathy due to hyperemesis gravidarum: Clinical and magnetic resonance imaging characteristics. J. Postgrad. Med..

[84] Frank L.L. (2015). Thiamin in Clinical Practice. J. Parenter. Enter. Nutr..

[85] Kareem O., Nisar S., Tanvir M., Muzaffer U., Bader G.N. (2023). Thiamine deficiency in pregnancy and lactation: Implications and present perspectives. Front. Nutr..

[86] Di Gangi S., Gizzo S., Patrelli T.S., Saccardi C., D’antona D., Nardelli G.B. (2012). Wernicke’s encephalopathy complicating hyperemesis gravidarum: From the background to the present. J. Matern. Neonatal Med..

[87] Finglas P.M. (2000). Dietary Reference intakes for thiamin, riboflavin, niacin, vitamin B6, folate, vitamin B12, pantothenic acid, biotin and choline. Trends Food Sci. Technol..

[88] Jans G., Matthys C., Bogaerts A., Lannoo M., Verhaeghe J., Van der Schueren B., Devlieger R. (2015). Maternal Micronutrient Deficiencies and Related Adverse Neonatal Outcomes after Bariatric Surgery: A Systematic Review. Adv. Nutr. Int. Rev. J..

[89] Kröll D., Laimer M., Borbély Y.M., Laederach K., Candinas D., Nett P.C. (2016). Wernicke Encephalopathy: A Future Problem Even After Sleeve Gastrectomy? A Systematic Literature Review. Obes. Surg..

[90] Ngene N.C., Moodley J. (2016). Clinical awareness for health care professionals: Fatal encephalopathy complicating persistent vomiting in pregnancy. S. Afr. Med. J..

[91] Bahardoust M., Eghbali F., Shahmiri S.S., Alijanpour A., Yarigholi F., Valizadeh R., Madankan A., Pouraskari A.B., Ashtarinezhad B., Farokhi H. (2022). B1 Vitamin Deficiency After Bariatric Surgery, Prevalence, and Symptoms: A Systematic Review and Meta-analysis. Obes. Surg..

[92] Saab R.O., El Khoury M.I., Jabbour R.A. (2013). Wernicke encephalopathy after Roux-en-Y gastric bypass and hyperemesis gravidarum. Surg. Obes. Relat. Dis..

[93] Holick M.F., Binkley N.C., Bischoff-Ferrari H.A., Gordon C.M., Hanley D.A., Heaney R.P., Murad M.H., Weaver C.M. (2011). Evaluation, Treatment, and Prevention of Vitamin D Deficiency: An Endocrine Society Clinical Practice Guideline. Med. J. Clin. Endocrinol. Metab..

[94] Wagner C.L., Hollis B.W. (2018). The Implications of Vitamin D Status During Pregnancy on Mother and her Developing Child. Front. Endocrinol..

[95] Bodnar L.M., Catov J.M., Simhan H.N., Holick M.F., Powers R.W., Roberts J.M. (2007). Maternal Vitamin D Deficiency Increases the Risk of Preeclampsia. J. Clin. Endocrinol. Metab..

[96] Wei S.-Q., Qi H.-P., Luo Z.-C., Fraser W.D. (2013). Maternal vitamin D status and adverse pregnancy outcomes: A systematic review and meta-analysis. J. Matern.-Fetal Neonatal Med..

[97] Hu K.-L., Zhang C.-X., Chen P., Zhang D., Hunt S. (2022). Vitamin D Levels in Early and Middle Pregnancy and Preeclampsia, a Systematic Review and Meta-Analysis. Nutrients.

[98] Perez-Ferre N., Torrejon M.J., Fuentes M., Fernandez M.D., Ramos A., Bordiu E., del Valle L., Rubio M.A., Bedia A.R., Montañez C. (2012). Association of Low Serum 25-Hydroxyvitamin D Levels in Pregnancy with Glucosehomeostasis and Obstetric And Newborn Outcomes. Endocr. Pract..

[99] Switzer N.J., Marcil G., Prasad S., Debru E., Church N., Mitchell P., Billington E.O., Gill R.S. (2017). Long-term hypovitaminosis D and secondary hyperparathyroidism outcomes of the Roux-en-Y gastric bypass: A systematic review. Obes. Rev..

[100] Chakhtoura M.T., Nakhoul N., Akl E.A., Mantzoros C.S., El Hajj Fuleihan G.A. (2016). Guidelines on vitamin D replacement in bariatric surgery: Identification and systematic appraisal. Metabolism.

[101] Medeiros M., Matos A.C., Pereira S.E., Saboya C., Ramalho A. (2016). Vitamin D and its relation with ionic calcium, parathyroid hormone, maternal and neonatal characteristics in pregnancy after roux-en-Y gastric bypass. Arch. Gynecol. Obstet..

[102] Cruz S., de Matos A.C., da Cruz S.P., Pereira S., Saboya C., Ramalho A. (2018). Maternal Anthropometry and Its Relationship with the Nutritional Status of Vitamin D, Calcium, and Parathyroid Hormone in Pregnant Women after Roux-en-Y Gastric Bypass. Obes. Surg..

[103] World Health Organization (2009). Global Prevalence of Vitamin A Deficiency in Populations at Risk 1995–2005: WHO Global Database on Vitamin A Deficiency.

[104] Snoek K., van de Woestijne N., Willemsen S., Klaassen R., Galjaard S., Laven J., Steegers-Theunissen R., Schoenmakers S. (2022). The Impact of Preconception Gastric Bypass Surgery on Maternal Micronutrient Status before and during Pregnancy: A Retrospective Cohort Study in the Netherlands between 2009 and 2019. Nutrients.

[105] OMS (2011). Concentraciones en suero de retinol para establecer la prevalencia de la carencia de vitamina A a escala poblacional. Sistema de Información Nutricional Sobre Vitaminas y Minerales.

[106] Rubio M.A., Cuesta M., Pelaz L., Pérez C., Torrejón M.J., Cabrerizo L., Matía P., Pérez-Ferre N., Sánchez-Pernaute A., Torres A. (2014). Fat-soluble vitamin deficiencies after bariatric surgery could be misleading if they are not appropriately adjusted. Nutr. Hosp..

[107] Rayman M.P., Bode P., Redman C.W.G. (2003). Low selenium status is associated with the occurrence of the pregnancy disease preeclampsia in women from the United Kingdom. Am. J. Obstet. Gynecol..

[108] Oliveira J.M., Michelazzo F.B., Stefanello J., Rondó P.H. (2008). Influence of iron on vitamin A nutritional status. Nutr. Rev..

[109] WHO (2011). Guideline: Vitamin A Supplementation in Pregnant Women.

[110] McCauley M.E., van den Broek N., Dou L., Othman M. (2015). Vitamin A supplementation during pregnancy for maternal and newborn outcomes. Cochrane Database Syst. Rev..

[111] EFSA NDA Panel (2015). Scientific Opinion on Dietary Reference Values for biotin. EFSA J..

[112] Slater G.H., Ren C.J., Siegel N., Williams T., Barr D., Wolfe B., Dolan K., A Fielding G. (2004). Serum fat-soluble vitamin deficiency andabnormal calcium metabolism after malabsorptivebariatric surgery. J. Gastrointest. Surg..

[113] Eckert M.J., Perry J.T., Sohn V.Y., Boden J., Martin M.J., Rush R.M., Steele S.R. (2010). Incidence of low vitamin A levels and ocular symptoms after Roux-en-Y gastric bypass. Surg. Obes. Relat. Dis..

[114] Pereira S., Saboya C., Chaves G., Ramalho A. (2009). Class III Obesity and its Relationship with the Nutritional Status of Vitamin A in Pre- and Postoperative Gastric Bypass. Obes. Surg..

[115] Vales Montero M., Chavarría Cano B., Martínez Ginés M.L., Díaz Otero F., Velázquez Pérez J.M., Cuerda Compes M.C., Bretón Lesmes I. (2016). Deficiencia clínica de vitamina A tras bypass gástrico: Descripción de un caso clínico y revisión de la literatura. Nutr. Hosp..

[116] Ocón J., Cabrejas C., Altemir J., Moros M. (2012). Phrynoderma. J. Parenter. Enter. Nutr..

[117] Fok J.S., Li J.Y.Z., Yong T.Y. (2012). Visual deterioration caused by vitamin A deficiency in patients after bariatric surgery. Eat. Weight. Disord.—Stud. Anorexia, Bulim. Obes..

[118] Ramos-Leví A.M., Pérez-Ferre N., Sánchez-Pernaute A., García A.J.T., Herrera M.A.R. (2013). Torres Garcia, and M. a Rubio Herrera, “Severe vitamin A deficiency after malabsortive bariatric surgery. Nutr. Hosp..

[119] Stroh C., Weiher C., Hohmann U., Meyer F., Lippert H., Manger T. (2009). Vitamin A Deficiency (VAD) After a Duodenal Switch Procedure: A Case Report. Obes. Surg..

[120] Chagas C.B., Saunders C., Pereira S., Silva J., Saboya C., Ramalho A. (2012). Vitamin A Deficiency in Pregnancy: Perspectives after Bariatric Surgery. Obes. Surg..

[121] Huerta S., Rogers L.M., Li Z., Heber D., Liu C., Livingston E.H. (2002). Vitamin A deficiency in a newborn resulting from maternal hypovitaminosis A after biliopancreatic diversion for the treatment of morbid obesity. Am. J. Clin. Nutr..

[122] Smets K.J., Barlow T., Vanhaesebrouck P. (2006). Maternal vitamin A deficiency and neonatal microphthalmia: Complications of biliopancreatic diversion?. Eur. J. Pediatr..

[123] Breton J.O., Sallan L. (2010). Maternal and neonatal complications in a pregnant woman with biliopancreatic diversion. [Spanish] TT—Complicaciones maternas y neonatales en una mujer gestante con derivacion biliopancreatica. Nutr. Hosp. Organo Of. Soc. Esp. Nutr. Parenter. Y Enter..

[124] Mackie F.L., Cooper N.S., Whitticase L.J., Smith A., Martin W.L., Cooper S.C. (2018). Vitamin A and micronutrient deficiencies post-bariatric surgery: Aetiology, complications and management in a complex multiparous pregnancy. Eur. J. Clin. Nutr..

[125] Cruz S., Matos A., Da Cruz S.P., Pereira S., Saboya C., Ramalho A. (2017). Relationship between the Nutritional Status of Vitamin A per Trimester of Pregnancy with Maternal Anthropometry and Anemia after Roux-en-Y Gastric Bypass. Nutrients.

[126] Hazart J., Le Guennec D., Accoceberry M., Lemery D., Mulliez A., Farigon N., Lahaye C., Miolanne-Debouit M., Boirie Y. (2017). Maternal Nutritional Deficiencies and Small-for-Gestational-Age Neonates at Birth of Women Who Have Undergone Bariatric Surgery. J. Pregnancy.

[127] Niki E., Traber M.G. (2012). A History of Vitamin E. Ann. Nutr. Metab..

[128] Traber M.G. (2014). Vitamin E Inadequacy in Humans: Causes and Consequences. Adv. Nutr. Int. Rev. J..

[129] Institute of Medicine, Food and Nutrition Board, Panel on Dietary Antioxidants and Related Compounds, Subcommittee on Upper Reference Levels of Nutrients, Subcommittee on Interpretation and Uses of Dietary Reference Intakes, Standing Committee on the Scientific Evaluation of Dietary Reference Intakes (2000). Dietary Reference Intakes for Vitamin C, Vitamin E, Selenium, and Carotenoids.

[130] Chen K., Zhang X., Wei X.-P., Qu P., Liu Y.-X., Li T.-Y. (2009). Antioxidant vitamin status during pregnancy in relation to cognitive development in the first two years of life. Early Hum. Dev..

[131] Koscik R.L., Lai H.J., Laxova A., Zaremba K.M., Kosorok M.R., Douglas J.A., Rock M.J., Splaingard M.L., Farrell P.M. (2005). Preventing early, prolonged vitamin E deficiency: An opportunity for better cognitive outcomes via early diagnosis through neonatal screening. J. Pediatr..

[132] Hovdenak N., Haram K. (2012). Influence of mineral and vitamin supplements on pregnancy outcome. Eur. J. Obstet. Gynecol. Reprod. Biol..

[133] Sitrin M.D., Lieberman F., Jensen W.E., Noronha A., Milburn C., Addington W. (1987). Vitamin E Deficiency and Neurologic Disease in Adults with Cystic Fibrosis. Ann. Intern. Med..

[134] Wilson R.D., Audibert F., Brock J.-A., Carroll J., Cartier L., Gagnon A., Johnson J.-A., Langlois S., Murphy-Kaulbeck L., Okun N. (2015). Pre-conception Folic Acid and Multivitamin Supplementation for the Primary and Secondary Prevention of Neural Tube Defects and Other Folic Acid-Sensitive Congenital Anomalies. J. Obstet. Gynaecol. Can..

[135] Bastos Maia S., Rolland Souza A.S., Costa Caminha M.d.F., Lins da Silva S., Callou Cruz R.d.S.B.L., Carvalho dos Santos C., Batista Filho M. (2019). Vitamin A and Pregnancy: A Narrative Review. Nutrients.

[136] Harshman S.G., Saltzman E., Booth S.L. (2014). Vitamin K. Curr. Opin. Clin. Nutr. Metab. Care.

[137] Pearson D.A. (2007). Bone Health and Osteoporosis: The Role of Vitamin K and Potential Antagonism by Anticoagulants. Nutr. Clin. Pract..

[138] Shahrook S., Ota E., Hanada N., Sawada K., Mori R. (2018). Vitamin K supplementation during pregnancy for improving outcomes: A systematic review and meta-analysis. Sci. Rep..

[139] Homan J., Ruinemans-Koerts J., Aarts E., Janssen I.M., Berends F.J., de Boer H. (2016). Management of vitamin K deficiency after biliopancreatic diversion with or without duodenal switch. Surg. Obes. Relat. Dis..

[140] Van Mieghem T., Van Schoubroeck D., Depiere M., Debeer A., Hanssens M. (2008). Fetal Cerebral Hemorrhage Caused by Vitamin K Deficiency After Complicated Bariatric Surgery. Obstet. Gynecol..

[141] Bersani I., De Carolis M.P., Salvi S., Zecca E., Romagnoli C., De Carolis S. (2011). Maternal–neonatal vitamin K deficiency secondary to maternal biliopancreatic diversion. Blood Coagul. Fibrinolysis.

[142] Jans G., Guelinckx I., Voets W., Galjaard S., Van Haard P.M., Vansant G.M., Devlieger R. (2014). Vitamin K1 monitoring in pregnancies after bariatric surgery: A prospective cohort study. Surg. Obes. Relat. Dis..

[143] Aron-Wisnewsky J., O Verger E., Bounaix C., Dao M.C., Oppert J.-M., Bouillot J.-L., Chevallier J.-M., Clément K. (2016). Nutritional and Protein Deficiencies in the Short Term following Both Gastric Bypass and Gastric Banding. PLoS ONE.

[144] DeLoughery T.G. (2017). Iron Deficiency Anemia. Med. Clin. N. Am..

[145] Aigner E., Feldman A., Datz C. (2014). Obesity as an Emerging Risk Factor for Iron Deficiency. Nutrients.

[146] Hamamy H., Alwan N.A. (2015). Maternal Iron Status in Pregnancy and Long-Term Health Outcomes in the Offspring. J. Pediatr. Genet..

[147] Spanish Ministry of Health (2014). Grupo de Trabajo de la Guía de Práctica Clínica de Atención en el Embarazo y Puerperio.

[148] Breymann C. (2005). Iron deficiency and anaemia in pregnancy: Modern aspects of diagnosis and therapy. Eur. J. Obstet. Gynecol. Reprod. Biol..

[149] World Health Organization (2012). Guideline: Daily Iron and Folic Acid Supplementation in Pregnant Women.

[150] Muñoz M., Rosado E.L. (2009). Iron Deficiency and Anaemia in Bariatric Surgical patients: Causes, Diagnosis and Proper Management. Nutr. Hosp..

[151] Jericó C., Bretón I., de Gordejuela A.G.R., de Oliveira A.C., Rubio M., Tinahones F.J., Vidal J., Vilarrasa N. (2016). Diagnóstico y tratamiento del déficit de hierro, con o sin anemia, pre y poscirugía bariátrica. Endocrinol. Y Nutr..

[152] Steenackers N., Van der Schueren B., Mertens A., Lannoo M., Grauwet T., Augustijns P., Matthys C. (2018). Iron deficiency after bariatric surgery: What is the real problem?. Proc. Nutr. Soc..

[153] Nomura R.M.Y., Dias M.C.G., Igai A.M.K., Paiva L.V., Zugaib M. (2011). Anemia During Pregnancy after Silastic Ring Roux-en-Y Gastric Bypass: Influence of Time to Conception. Obes. Surg..

[154] Falcone V., Stopp T., Feichtinger M., Kiss H., Eppel W., Husslein P.W., Prager G., Göbl C.S. (2018). Pregnancy after bariatric surgery: A narrative literature review and discussion of impact on pregnancy management and outcome. BMC Pregnancy Childbirth.

[155] Hezelgrave N.L., Oteng-Ntim E. (2011). Pregnancy after Bariatric Surgery: A Review. J. Obes..

[156] Dalfrà M.G., Busetto L., Chilelli N.C., Lapolla A. (2012). Pregnancy and foetal outcome after bariatric surgery: A review of recent studies. J. Matern. Neonatal Med..

[157] Gröber U., Schmidt J., Kisters K. (2015). Magnesium in Prevention and Therapy. Nutrients.

[158] De Baaij J.H.F., Hoenderop J.G.J., Bindels R.J. (2015). Magnesium in Man: Implications for Health and Disease. Physiol. Rev..

[159] Costello R.B., Elin R.J., Rosanoff A., Wallace T.C., Guerrero-Romero F., Hruby A., Lutsey P.L., Nielsen F.H., Rodriguez-Moran M., Song Y. (2016). Perspective: The Case for an Evidence-Based Reference Interval for Serum Magnesium: The Time Has Come. Adv. Nutr..

[160] Lager S., Powell T.L. (2012). Regulation of Nutrient Transport across the Placenta. J. Pregnancy.

[161] Amaral L.M., Wallace K., Owens M., LaMarca B. (2017). Pathophysiology and Current Clinical Management of Preeclampsia. Curr. Hypertens. Rep..

[162] Holcberg G., Sapir O., Hallak M., Alaa A., Shorok H.-Y., David Y., Katz M., Huleihel M. (2004). Selective Vasodilator Effect of Magnesium Sulfate in Human Placenta. Am. J. Reprod. Immunol..

[163] Dhariwal N.K., Lynde G.C. (2017). Update in the Management of Patients with Preeclampsia. Anesthesiol. Clin..

[164] Dalton L.M., Fhloinn D.M.N., Gaydadzhieva G.T., Mazurkiewicz O.M., Leeson H., Wright C.P. (2016). Magnesium in pregnancy. Nutr. Rev..

[165] Makrides M., Crosby D.D., Shepherd E., A Crowther C. (2014). Magnesium supplementation in pregnancy. Cochrane Database Syst. Rev..

[166] Shepherd E., Salam R.A., Manhas D., Synnes A., Middleton P., Makrides M., Crowther C.A. (2019). Antenatal magnesium sulphate and adverse neonatal outcomes: A systematic review and meta-analysis. PLOS Med..

[167] Gimenes J.C., Nicoletti C.F., de Souza Pinhel M.A., de Oliveira B.A.P., Júnior W.S., Marchini J.S., Nonino C.B. (2017). Pregnancy After Roux en Y Gastric Bypass: Nutritional and Biochemical Aspects. Obes. Surg..

[168] Trumbo P., A Yates A., Schlicker S., Poos M. (2001). Dietary Reference Intakes. J. Am. Diet. Assoc..

[169] Prasad A.S. (2013). Discovery of Human Zinc Deficiency: Its Impact on Human Health and Disease. Adv. Nutr. Int. Rev. J..

[170] Roohani N., Hurrell R., Kelishadi R., Schulin R. (2013). Zinc and its importance for human health: An integrative review. J. Res. Med. Sci..

[171] Wilson R.L., Grieger J.A., Bianco-Miotto T., Roberts C.T. (2016). Association between Maternal Zinc Status, Dietary Zinc Intake and Pregnancy Complications: A Systematic Review. Nutrients.

[172] Walker J.B., Houseman J., Seddon L., McMullen E., Tofflemire K., Mills C., Corriveau A., Weber J.-P., LeBlanc A., Walker M. (2006). Maternal and umbilical cord blood levels of mercury, lead, cadmium, and essential trace elements in Arctic Canada. Environ. Res..

[173] King J.C. (2000). Determinants of maternal zinc status during pregnancy. Am. J. Clin. Nutr..

[174] Hanachi P., Norrozi M., Moosavi R.M. (2014). The Correlation of Prenatal Zinc Concentration and Deficiency with Anthropometric Factors. J. Fam. Reprod. Health.

[175] Shen P.-J., Gong B., Xu F.-Y., Luo Y. (2015). Four trace elements in pregnant women and their relationships with adverse pregnancy outcomes. Eur. Rev. Med. Pharmacol. Sci..

[176] Atazadegan M.A., Heidari-Beni M., Riahi R., Kelishadi R. (2022). Association of selenium, zinc and copper concentrations during pregnancy with birth weight: A systematic review and meta-analysis. J. Trace Elem. Med. Biol..

[177] Rojas P., Gosch M., Basfi-Fer K., Carrasco F., Codoceo J., Inostroza J., Valencia A., Adjemian D., Rojas J., Díaz E. (2011). Alopecia in women with severe and morbid obesity who undergo bariatric surgery. Nutr. Hosp..

[178] Chagas C., Saunders C., Pereira S., Silva J., Saboya C., Ramalho A. (2016). Vitamin A status and its relationship with serum zinc concentrations among pregnant women who have previously undergone Roux-en-Y gastric bypass. Int. J. Gynecol. Obstet..

[179] Ducarme G., Planche L., Abet E., du Roure V.D., Ducet-Boiffard A. (2021). A Prospective Study of Association of Micronutrients Deficiencies during Pregnancy and Neonatal Outcome among Women after Bariatric Surgery. J. Clin. Med..

[180] Scheiber I.F., Mercer J.F., Dringen R. (2014). Metabolism and functions of copper in brain. Prog. Neurobiol..

[181] Tiffany-Castiglioni E., Hong S., Qian Y. (2011). Copper handling by astrocytes: Insights into neurodegenerative diseases. Int. J. Dev. Neurosci..

[182] Altarelli M., Ben-Hamouda N., Schneider A., Berger M.M. (2019). Copper Deficiency: Causes, Manifestations, and Treatment. Nutr. Clin. Pract..

[183] Uriu-Adams J.Y., Scherr R.E., Lanoue L., Keen C.L. (2010). Influence of copper on early development: Prenatal and postnatal considerations. Biofactors.

[184] Zhang Z., Yuan E., Liu J., Lou X., Jia L., Li X., Zhang L. (2013). Gestational age-specific reference intervals for blood copper, zinc, calcium, magnesium, iron, lead, and cadmium during normal pregnancy. Clin. Biochem..

[185] Alebic-Juretic A., Frkovic A. (2005). Plasma copper concentrations in pathological pregnancies. J. Trace Elem. Med. Biol..

[186] Keen C.L., Uriu-Hare J.Y., Hawk S.N., Jankowski M., Daston G.P., Kwik-Uribe C.L., Rucker R.B. (1998). Effect of copper deficiency on prenatal development and pregnancy outcome. Am. J. Clin. Nutr..

[187] Saltzman E., Karl J.P. (2013). Nutrient Deficiencies After Gastric Bypass Surgery. Annu. Rev. Nutr..

[188] Gletsu-Miller N., Broderius M., Frediani J.K., Zhao V.M., Griffith D.P., Davis S.S., Sweeney J.F., Lin E., Prohaska J.R., Ziegler T.R. (2012). Incidence and prevalence of copper deficiency following roux-en-y gastric bypass surgery. Int. J. Obes..

[189] Kumar P., Hamza N., Madhok B., De Alwis N., Sharma M., Miras A.D., Mahawar K.K. (2016). Copper Deficiency after Gastric Bypass for Morbid Obesity: A Systematic Review. Obes. Surg..

[190] Balsa J.A., Botella-Carretero J.I., Gómez-Martín J.M., Peromingo R., Arrieta F., Santiuste C., Zamarrón I., Vázquez C. (2011). Copper and Zinc Serum Levels after Derivative Bariatric Surgery: Differences between Roux-en-Y Gastric Bypass and Biliopancreatic Diversion. Obes. Surg..

[191] Btaiche I.F., Yeh A.Y., Wu I.J., Khalidi N. (2011). Neurologic Dysfunction and Pancytopenia Secondary to Acquired Copper Deficiency Following Duodenal Switch. Nutr. Clin. Pract..

[192] Griffith D.P., Liff D.A., Ziegler T.R., Esper G.J., Winton E.F. (2009). Acquired Copper Deficiency: A Potentially Serious and Preventable Complication Following Gastric Bypass Surgery. Obesity.

[193] Rowin J., Lewis S.L. (2005). Copper deficiency myeloneuropathy and pancytopenia secondary to overuse of zinc supplementation. J. Neurol. Neurosurg. Psychiatry.

[194] Pieczyńska J., Grajeta H. (2014). The role of selenium in human conception and pregnancy. J. Trace Elem. Med. Biol..

[195] Combs J.G.F. (2015). Biomarkers of Selenium Status. Nutrients.

[196] EFSA Panel on Dietetic Products, Nutrition and Allergies (NDA) (2014). Scientific Opinion on Dietary Reference Values for selenium. EFSA J..

[197] Xu M., Guo D., Gu H., Zhang L., Lv S. (2016). Selenium and Preeclampsia: A Systematic Review and Meta-analysis. Biol. Trace Elem. Res..

[198] Hamdan H.Z., Hamdan S.Z., Adam I. (2023). Association of Selenium Levels with Preeclampsia: A Systematic Review and Meta-analysis. Biol. Trace Elem. Res..

[199] Álvarez S.I., Castañón S.G., Ruata M.L.C., Aragüés E.F., Terraz P.B., Irazabal Y.G., González E.G., Rodríguez B.G. (2007). Updating of normal levels of copper, zinc and selenium in serum of pregnant women. J. Trace Elem. Med. Biol..

[200] Bermúdez L., García-Vicent C., López J., Torró M.I., Lurbe E. (2015). Assessment of ten trace elements in umbilical cord blood and maternal blood: Association with birth weight. J. Transl. Med..

[201] Boldery R., Fielding G., Rafter T., Pascoe A.L., Scalia G.M. (2007). Nutritional Deficiency of Selenium Secondary to Weight Loss (Bariatric) Surgery Associated with Life-Threatening Cardiomyopathy. Heart Lung Circ..

[202] Massoure P.-L., Camus O., Fourcade L., Simon F. (2017). Bilateral leg oedema after bariatric surgery: A selenium-deficient cardiomyopathy. Obes. Res. Clin. Pract..

[203] Alasfar F., Ben-Nakhi M., Khoursheed M., Kehinde E.O., Alsaleh M. (2011). Selenium Is Significantly Depleted Among Morbidly Obese Female Patients Seeking Bariatric Surgery. Obes. Surg..

[204] Freeth A., Prajuabpansri P., Victory J.M., Jenkins P. (2012). Assessment of Selenium in Roux-en-Y Gastric Bypass and Gastric Banding Surgery. Obes. Surg..

[205] Papamargaritis D., Aasheim E.T., Sampson B., le Roux C.W. (2015). Copper, selenium and zinc levels after bariatric surgery in patients recommended to take multivitamin-mineral supplementation. J. Trace Elem. Med. Biol..

[206] Eltweri A.M., Bowrey D.J., Sutton C.D., Graham L., Williams R.N. (2013). An audit to determine if vitamin b12 supplementation is necessary after sleeve gastrectomy. Springerplus.

[207] Shahmiri S.S., Eghbali F., Ismaeil A., Gholizadeh B., Khalooeifard R., Valizadeh R., Rokhgireh S., Kermansaravi M. (2022). Selenium Deficiency After Bariatric Surgery, Incidence and Symptoms: A Systematic Review and Meta-Analysis. Obes. Surg..

[208] Hatch-McChesney A., Lieberman H.R. (2022). Iodine and Iodine Deficiency: A Comprehensive Review of a Re-Emerging Issue. Nutrients.

[209] Nazeri P., Shariat M., Azizi F. (2021). Effects of iodine supplementation during pregnancy on pregnant women and their offspring: A systematic review and meta-analysis of trials over the past 3 decades. Eur. J. Endocrinol..

[210] Michalaki M., Volonakis S., Mamali I., Kalfarentzos F., Vagenakis A.G., Markou K.B. (2014). Dietary iodine absorption is not influenced by malabsorptive bariatric surgery. Obes Surg..

[211] Lecube A., Zafon C., Gromaz A., Fort J.M., Caubet E., Baena J.A., Tortosa F. (2014). Iodine Deficiency Is Higher in Morbid Obesity in Comparison with Late after Bariatric Surgery and Non-obese Women. Obes. Surg..

[212] Manousou S., Carlsson L.M.S., Eggertsen R., Hulthén L., Jacobson P., Landin-Wilhelmsen K., Trimpou P., Svensson P.-A., Nyström H.F. (2018). Iodine Status After Bariatric Surgery—A Prospective 10-Year Report from the Swedish Obese Subjects (SOS) Study. Obes. Surg..

[213] Cheah S., Gao Y., Mo S., Rigas G., Fisher O., Chan D.L., Chapman M.G., Talbot M.L. (2022). Fertility, pregnancy and post partum management after bariatric surgery: A narrative review. Med. J. Aust..

